# A High‐Efficiency Yet Sustainable Fresh‐Keeping Strategy Inspired by Calyx of *Physalis peruviana*

**DOI:** 10.1002/EXP.20250786

**Published:** 2026-07-29

**Authors:** Yue Zhao, Chengbang Lu, Xiaopeng Bai, Shangyi Wu, Zeyu Wang, Yuqin Hu, Binxin Liang, Danlei Sun, Yunxiang Zhang, Yingning He, Yi Yang, Shengwen Duan, Taolin Sun, Xinjie Wang, Weijie Lan, Hong Zhang, Yang Luo, Xiangyu Liang

**Affiliations:** ^1^ Department of Laboratory Medicine Chongqing Center for Clinical Laboratory School of Medicine Chongqing Academy of Medical Sciences Chongqing General Hospital Chongqing University Chongqing China; ^2^ Institute of Bast Fiber Crops & Center of Southern Economic Crops Chinese Academy of Agricultural Sciences Changsha China; ^3^ Agricultural Genomics Institute at Shenzhen Chinese Academy of Agricultural Sciences Shenzhen China; ^4^ Department of Biomedical Engineering National University of Singapore Singapore Singapore; ^5^ School of Flexible Electronics Sun Yat‐Sen University Shenzhen China China; ^6^ South China Advanced Institute for Soft Matter Science and Technology South China University of Technology Guangzhou China; ^7^ Department of Food Science and Engineering Jilin Agricultural University Jilin China; ^8^ School of Physics and Optoelectronic Engineering Xiangtan University Hunan China; ^9^ State Key Laboratory of Molecular Engineering of Polymers Department of Macromolecular Science Fudan University Shanghai China; ^10^ Department of Food Science & Technology National University of Singapore Singapore Singapore; ^11^ College of Food Science and Technology Nanjing Agricultural University Nanjing China

**Keywords:** antimicrobial, bioinspired structure, electrospun fibers, food safety, sustainability

## Abstract

To address the dual challenges of foodborne illness and plastic pollution, we present a sprayable, calyx‐inspired food wrap engineered for safety and sustainability. Utilizing handheld electrospraying, zeolite‐doped polycaprolactone forms a multi‐scale network mimicking *Physalis peruviana*’s protective structure. This polycaprolactone/chitosan/zeolite wrap provides a health‐critical barrier, achieving >95% inhibition of tobacco mosaic virus and >92% antibacterial efficacy against pathogens such as *Escherichia coli* and *Staphylococcus aureus*. Additionally, it maintains high breathability (∼1500 g·m^−2^·day^−1^) to prevent food spoilage. Crucially, the wrap is non‐toxic and fully biodegradable within 180 days, eliminating microplastic risks. In real‐world validation, it extended the shelf life of perishable fruits by >7 days without quality loss, reducing food waste. Life cycle assessment confirmed an 84.97% reduction in CO_2_ emissions compared with conventional wraps, directly contributing to environmental health. This technology offers a scalable solution for sustainable nutrition security through a user‐friendly application, enhanced food safety, and rapid environmental degradation.

## Graphical abstract

This study presents a sprayable, calyx‐inspired food wrap engineered for safety and sustainability using handheld electrospraying. The wrap forms a health‐critical protective barrier with high antiviral and antibacterial efficacy, excellent breathability, and long‐term fresh‐keeping performance. A life cycle assessment confirms a significant reduction in CO_2_ emissions, directly contributing to environmental sustainability.

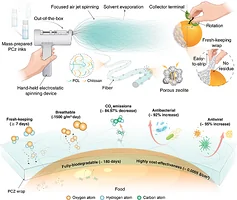

## 1 Introduction

Inadequate preservation and storage methods result in approximately 1.3 billion tons of global food waste annually, which equals one‐third of the global food supply [1]. This incurs substantial economic losses (e.g., an estimated $165 billion annually in the US [2] and approximately half of food wasted each year in the EU [3]) and disproportionately affects highly perishable fruits and vegetables, with 20% to 50% lost globally throughout the supply chain [4]. In China, post‐harvest losses of fruits and vegetables remain high at 20%–30%, largely due to inadequate cold‐chain logistics and low‐performance packaging materials [5]. In contrast, developed countries like the U.S. have reduced losses to below 10% through advanced preservation technologies and cold‐chain coverage exceeding 90% [6]. With only about 35% cold‐chain utilization in China [7], there is an urgent need for sustainable packaging solutions to enhance freshness preservation and reduce waste. This wastage depletes valuable resources [8] and raises critical concerns regarding food safety, being linked to significant foodborne illnesses globally [9]. Consequently, mitigating food waste and ensuring food safety constitute pressing global challenges with profound socio‐economic and environmental impacts.

Addressing these challenges, prolonging food freshness is essential for reducing waste and ensuring safety [10]. Various methods, including packaging, wax coating, chemical preservatives, and cold storage, are available. Among them, packaging plays a crucial role by safeguarding against harmful factors [11]. While synthetic plastics like polyethylene (PE), polypropylene (PP), and polyethylene terephthalate (PET) are widely used for their mechanical and barrier properties [12], their non‐degradability leads to severe environmental pollution [13]. Biodegradable packaging is an environmentally friendly alternative [13, 14]. However, current biodegradable options often inadequately meet the requirements for effective long‐term preservation, suffering from suboptimal air/moisture permeability, limited antimicrobial/antiviral properties, and difficulty preserving opened food.

However, a core bottleneck restricting the commercial application of current active food packaging materials (e.g., bio‐based films, edible coatings, nanocomposites) is their insufficient mechanical performance [15]. This performance deficiency primarily arises from molecular structure defects, environmental responsiveness, and limitations in nano‐reinforcement. The future can be improved through innovations in material design (e.g., dynamic covalent crosslinking for self‐healing, surface modification to improve nanoparticle dispersibility, and multilayer structures to balance strength and toughness) [16], optimization of processing technologies (e.g., electrospinning and 3D printing), and functional integration (e.g., antimicrobial properties, mechanical synergy, and environmental adaptability) [17]. To achieve a better balance between the mechanical performance and functionalization of active packaging materials, future research could focus on strategies such as biomimetic design, smart responsiveness, and full life cycle sustainability.

Over millions of years of evolution, the fruit calyx (persistent calyx) has evolved over millions of years to protect fruits and extend their post‐harvest freshness [18]. For instance, a study showed the calyx prolongs the shelf life of *Physalis peruviana* from 11 to 24 days at room temperature [19]. Its unique structure, with veins and stomata, regulates gas exchange and acts as a physical barrier against pathogens, offering valuable insights for biomimetic fresh‐keeping technologies (Figure 1a,b).

**FIGURE 1:**
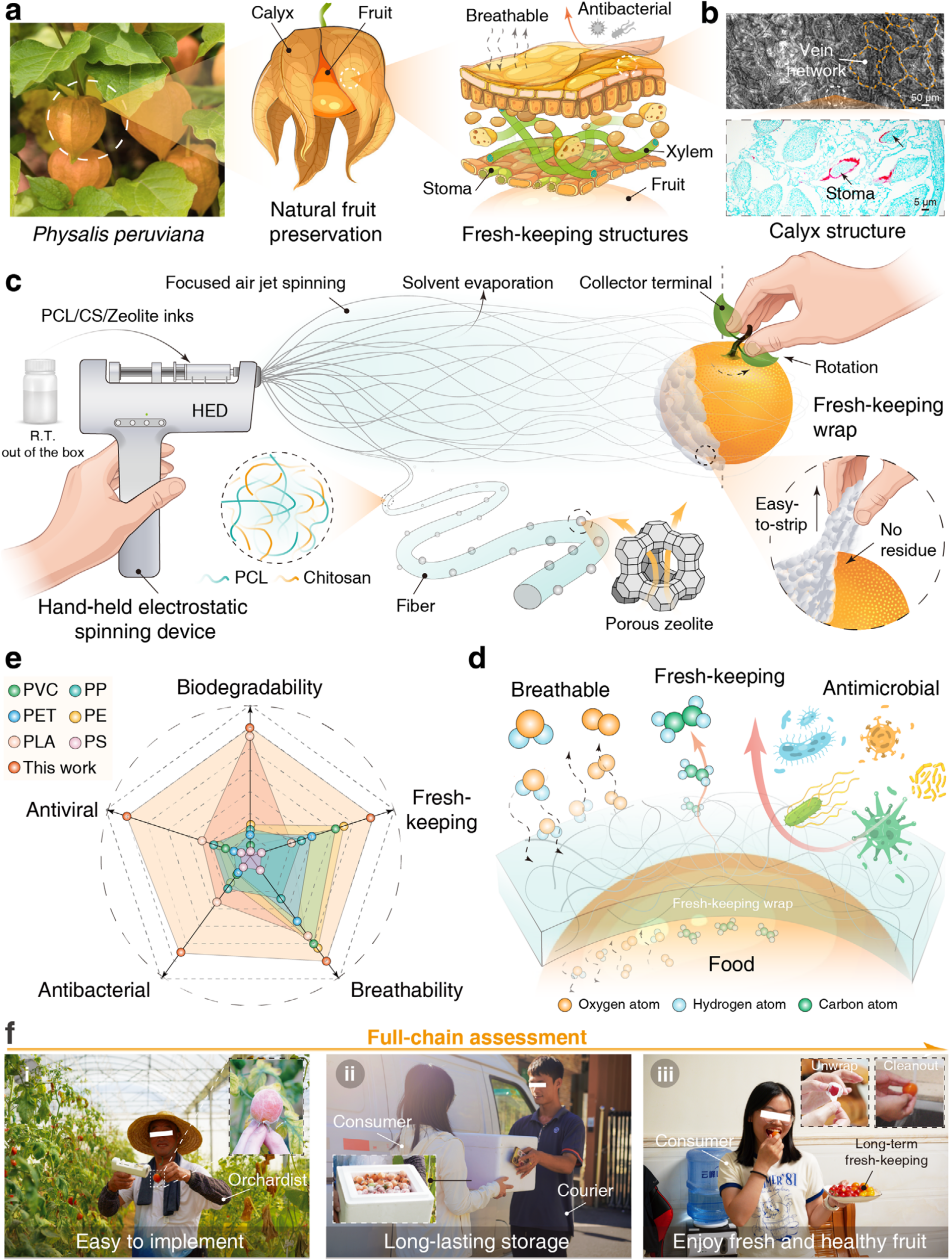
Design, fabrication, and application of PCZ wrap. (a) Schematic illustration of characteristics and microstructures of the calyx in *Physalis peruviana*. (b) Optical microscope observation (up, scale bar: 50 µm) and histological observation (down, scale bar: 5 µm) of the calyx, with the stoma highlighted by black arrows. (c) The fabrication process and multi‐scale components of PCZ wrap using a self‐developed hand‐held electro‐jetting device (HED) for ready‐to‐use fruit fresh‐keeping, showing the state of PCZ wrap on the fruit. (d) Schematic illustration of the fresh‐keeping mechanism of PCZ wrap, demonstrating its breathable and antimicrobial performances. (e) Summary and comparison between PCZ wrap and other commercial food wraps. (f) Practical full‐chain assessment of PCZ wrap from manufacturing to orchard implementation to consumer‐end usage to post‐use treatments.

Inspired by the unique structure‐function features of the *Physalis peruviana* calyx, we have developed a highly efficient, user‐friendly, and versatile strategy for producing fully biodegradable, breathable, antibacterial, and antiviral meshy wraps that are highly cost‐effective and ready‐to‐use, aimed at prolonging the shelf life of perishable fruits (Figure 1c). This strategy was enabled by our independently developed handheld electro‐jetting device (HED). Distinct from traditional industrial fabrication methods constrained by scale, HED's portability (weight = 350 g, power ≤ 50 W) facilitates the on‐site, immediate preparation of nanofiber films, addressing the needs of small‐scale farmers. Utilizing HED, we successfully integrated zeolite with high ethylene adsorption capabilities into a polycaprolactone/chitosan‐based polymer matrix to fabricate a polycaprolactone/chitosan/zeolite (PCZ) wrap featuring a multi‐scale network structure. Building upon these biological insights, the biomimetic design of the PCZ wrap transcended mere morphological replication of the calyx. Instead, it functionally emulates the calyx's protective mechanisms through a tripartite strategy (Figure 1d): (1) The microenvironmental regulation is facilitated by an electro‐jetted multi‐scale porous network, which ensures enhanced breathability and effective ethylene adsorption. (2) Additionally, a conformal chitosan‐based dual‐network coating serves as a physical barrier and offers antimicrobial protection through continuous surface coverage and intrinsic antibacterial properties. (3) Furthermore, mechanical compatibility is attained through a thin, flexible structure that adapts to the irregular surfaces of fruit without inflicting damage.

To validate this strategy, we selected two types of fruits (non‐climacteric and climacteric), specifically cherry (non‐climacteric) and tomatoes, bananas, and mangoes (climacteric), as evaluation models, recognizing their susceptibility to spoilage in the food supply chain. Compared to current petrochemical‐based polyvinyl chloride, PET, PP, PE, and polystyrene wraps, and even biodegradable polylactic acid (PLA) wrap on the global market, PCZ wrap demonstrated superior breathability, antibacterial, and antiviral properties, high cost‐effectiveness, and fully biodegradable properties (Figure 1e). This study has assessed the mass producibility, convenience, and effectiveness of the PCZ wrap across the entire supply chain (Figure 1f), from orchard production to transportation system to consumer‐end application. Our findings confirmed that PCZ wrap showcased a sustainable yet high‐efficiency preponderance for food fresh‐keeping. It will prospectively become the next‐generation food packaging alternative in addressing global food quality and safety issues.

## 2 Methods

### 2.1 Materials and Chemicals

Unless specified, the materials and chemicals used in this work were used without further purification. Polycaprolactone (PCL, average *M*_n_ ∼80 kDa) was purchased from Mackin (China). Chitosan (CS, from shrimp shells, deacetylated ≥75%) was purchased from Sigma‐Aldrich (Germany). Formic acid (FA, A.C.S. reagent, 98%) and acetic acid (AA, A.C.S. reagent, 99.7%) were purchased from Sigma‐Aldrich (Germany). Fresh tomatoes (variety: *Sweet 100*, weight: 80–150 g, origin: Italy), fresh cherries (variety: *Santina*, size: 24.6–26 mm, origin: Chile), bananas (variety: *Williams*, weight: 160–180 g, origin: Guangxi, China), mangos (variety: *Tainong No.1*, weight: 120–140 g, origin: Hainan, China), apples (variety: *Red Fuji*, weight: 180–195 g, origin: Shandong, China), and pears (variety: *Fragrant pear*, weight: 150–165 g, origin: Xinjiang, China) were purchased from local Chinese fruit markets (Table S1). The HED was specially designed and manufactured in cooperation with Qingdao Nuokang Environmental Protection Technology Co., Ltd. (Qingdao, China). Zeolites (*β*‐cage: a large cage, 11.6 Å; pores, 4.4 Å; length of a side, 6.6 Å) were purchased from Sigma‐Aldrich (Germany). Commercial cling wrap (aluminum foil, PE, and PLA) was purchased from Jiangsu Nantong Huitong Plastic Machinery Co., Ltd. (Jiangsu, China) and used as a control. *Physalis peruviana* was obtained by cultivating it in our laboratory.

### 2.2 Preparation of PCL/CS/ Zeolite (PCZ) Ink

To prepare PCZ wrap, we designed a precursor ink of PCL, chitosan (CS), and zeolite. The 2 wt.% CS solution was made by dissolving 2 g of CS in 50 mL of 90% aqueous acetic acid, followed by adding 6 g of PCL and stirring at 50°C until dissolved. Zeolites were added at 0, 0.5, and 1% mass ratios. The ink was delivered to the spinneret via a syringe pump, depositing fibers directly on the fruit epidermis, with collection time controlling the mass per unit surface area. Optimized parameters were 12% (w/v) PCL, 4% (w/v) CS, 0.5% (w/v) zeolite, and an ink flow rate of 7.5–10 mL·min^−1^.

### 2.3 Characteristic Analysis of Membrane Surface

Surface morphology and structural characteristics were analyzed using Scanning Electron Microscopy (SEM, Hitachi SU‐70). Samples were coated with platinum to enhance conductivity for high‐resolution imaging, typically performed at an accelerating voltage. Molecular structure and functional groups were determined using Fourier‐transform infrared spectroscopy (FT‐IR; Thermo‐iS50, USA), with spectra collected in the range for analysis of functional groups and chemical bonds. Water contact angle was measured using a goniometer (SDC‐100, SINDIN) at room temperature. A 3.5 µL droplet of distilled water was applied to the membrane surface, and angles were measured on both sides of the droplet. At least five measurements were taken at different positions on each sample and averaged to ensure statistical reliability.

### 2.4 Molecular Simulation of Ethylene Adsorption

The simulation of ethylene molecule adsorption was performed based on the Vienna Ab‐initio Simulation Package, using the Perdew–Burke–Ernzerhof function in the generalized gradient approximation to describe exchange‐correlation effects and the projective augmented wave method to handle nuclear‐valency interactions. The energy cutoff was set to 400 eV, and the structural optimization was completed with energy and force convergence set to 1.0 × 10^−5^ eV and 0.02 eV·Å^−1^. The Brillouin zone was sampled using a 2 × 2 × 3 K point, and dispersion interactions were described using Grimme's DFT‐D3 method. Adsorption energy (*E*_ads_) of ethylene molecules was calculated as follows: $E_{\mathrm{ads}}=E_{C_{2}H_{4}}-E_{-C_{2}H_{4}}-E_{\mathrm{sub}}$, where $E_{C_{2}H_{4}}$ and $E_{C_{2}H_{4}}$ represent the energies before and after adsorption, respectively, and $E_{\_\mathrm{sub}}$ is the energy of the clean surface. Electrostatic potentials (ESPs) were computed using Gaussian 09 with B3LYP and def2‐TZVP basis sets. Ethylene adsorption isotherms were measured at 77 K using an Autosorb 3B, with surface area and micropore volume determined by BET and Dubinin–Radushkevich models.

### 2.5 Measurement of Water Vapor and Air Permeability

Water vapor transmission rate (WVTR) of PCZ wrap was evaluated following the gravimetric method according to the national standard GB/T 12704.1‐2009. Tests were conducted under controlled temperature and relative humidity conditions. Samples were sealed onto cups containing deionized water and placed in a chamber with silica gel desiccant. The mass change was recorded periodically to determine the transmission rate. Air permeability (AP) was measured using a YG46E‐ІІІІ instrument. A sample area of 20 cm^2^ was tested under a pressure of 100 Pa, with results calculated and expressed in appropriate units.

### 2.6 Biodegradability Assessment

In the soil biodegradability test, 3 cm × 1 cm pieces of PE film and PCL/CS/Zeolite (0.5 wt.%) wrap were buried in petri dishes (*D*: 75 mm) filled with natural soil. Monthly biodegradation tests were conducted, with soil moisture maintained through daily watering. Samples were retrieved, photographed (Figure S1), and their residual mass was measured to calculate the degradation rate (%), as follows: $\mathrm{Degradation}\;\mathrm{rate}\;\left(\%\right)=\left(\frac{M_{0}-M_{t}}{M_{0}}\right)\times 100\%$, where *M*_0_ is the initial mass and *M*_t_ the residual mass after time *t*.

### 2.7 Evaluation of Antibacterial Activity

The PCZ wraps produced were submitted to the analysis of antimicrobial activity by disk diffusion, using two different bacteria: *Escherichia coli* (*E. coli*, ATCC25922) and *Staphylococcus aureus* (*S. aureus*, ATCC25923). The bacteria were recovered in brain heart infusion broth (37°C, 24 h) and diluted in saline solution (NaCl 9 g·L^−1^) until 0.5 McFarland (1–2 × 10^8^ UFC·mL^−1^). Disks of 6 mm in diameter were applied to petri dishes containing Mueller–Hinton agar, previously inoculated with the microorganisms. The plates with the disks were left at 37°C, and the inhibition zone was measured after 24 h.

### 2.8 Evaluation of Fruit Fresh‐Keeping Effect

To evaluate the fresh‐keeping efficacy of PCZ wrap, cherries, tomatoes, bananas, and mangoes were sourced from a local market, ensuring the selection of intact, mold‐free samples with similar size, weight, and color. After washing with deionized water for 30 min and air drying, the fruits were divided into blank and treatment groups, with the latter covered in PCZ wrap. The samples were stored at 25 ± 2°C with 50% humidity, and assessments were performed on days 0, 3, and 7, including measurements of weight loss, color stability, firmness, peroxidase (POD) enzyme activity, and reducing sugar content (RSC). Weight loss was monitored at specified intervals, and color changes were quantified using a colorimeter to record L* (brightness), a* (red/green), and b* (yellow/blue) values, with total color variation (∆*E*) calculated as follows: $\Delta E=\sqrt{{\left(L_{2}^{*}-L_{1}^{*}\right)}^{2}+{\left(a_{2}^{*}-a_{1}^{*}\right)}^{2}+{\left(b_{2}^{*}-b_{1}^{*}\right)}^{2}}$, where $L_{1}^{*}$, $a_{1}^{*}$, $b_{1}^{*}$ and $L_{2}^{*}$, $a_{2}^{*}$, $b_{2}^{*}$ are the *L*_ab_* values at the beginning and at a certain point in time, respectively. By calculating ΔE, the color change during storage can be assessed.

Firmness was assessed with a universal testing machine (AGS‐10kNXD) using an 8 cm length, 3 mm diameter probe at a speed of 1.5 mm·s^−1^. POD enzyme activity and RSC were determined by extracting 5 g of fruit in respective buffer solutions, homogenizing in an ice bath, and centrifuging at 12,000 × *g* for 30 min. The supernatants were analyzed using Solarbio ELISA kits (Beijing, Solarbio Life Sciences).

### 2.9 Life‐Cycle Assessment (LCA) and PCZ Wrap Model Evaluation

The life‐cycle assessment (LCA), following ISO 14040 standards [20], was conducted using OpenLCA 1.10.3 with two functional units: 1 kg of PCZ wrap and normalization by tensile strength. System boundaries included raw material production, transportation, and bioplastic manufacturing. An environmental impact model was built using LCA data, population, and 2021 per capita fruit consumption, assuming 1 kg of plastic wrap per 1 kg of fruit. The product life cycle model predicted market demand and assessed impacts such as global warming potential (GWP), acidification potential (AP), and terrestrial ecotoxicity potential (TETP). A comparative analysis with conventional plastic wrap highlighted the environmental benefits of PCZ wrap, ranking the top 15 countries with the highest potential improvements. R Studio and the rnaturalearth package were used for global data visualization. Code is available at: https://github.com/domelxb/PCZ‐wrap.

### 2.10 Metabolomics Analysis

Using vacuum freeze‐drying technology, place the cherry fruit tissues in a lyophilizer (Scientz‐100F), then grind (30 Hz, 1.5 min) the samples to powder form by using a grinder (MM 400, Retsch). Next, 50 mg of sample powder was weighed using an electronic balance (MS105DM), and 1200 µL of −20°C pre‐cooled 70% methanolic aqueous internal standard extract (less than 50 mg added at the rate of 1200 µL of extractant per 50 mg of sample) was added. The mixture was vortexed every 30 min for 30 s, a total of 6 times. After centrifugation (rotation speed 12,000 rpm, 3 min), the supernatant was aspirated, and the sample was filtered through a microporous membrane (0.22 µm pore size) and stored in the injection vial for UPLC‐MS/MS analysis [21].

## 3 Results

### 3.1 Design and Fabrication of PCZ Wrap

Based on the stable material system (Figure S2), we successfully fabricated a PCZ wrap with a network structure using a self‐developed hand‐held electro‐jetting device (HED). During the process, the precursor ink was jetted in a high‐voltage electric field, enabling rapid solvent evaporation (Figure S3) and facilitating the deposition of a homogeneous fibrous network. The matrix of the fibrous network was constructed via hydrogen bonding between PCL and CS, into which zeolite particles were evenly filled, forming a stabilized ternary compound system at room temperature. The resulting network exhibited a hierarchical porous structure formed by the deposition of multi‐layered fibers. This structure resembled the intersecting vein patterns found in a calyx, consistent with principles like Murray's law [20, 21]. This strategy demonstrated significant feasibility for batch production and practical application (Figure 2a).

**FIGURE 2:**
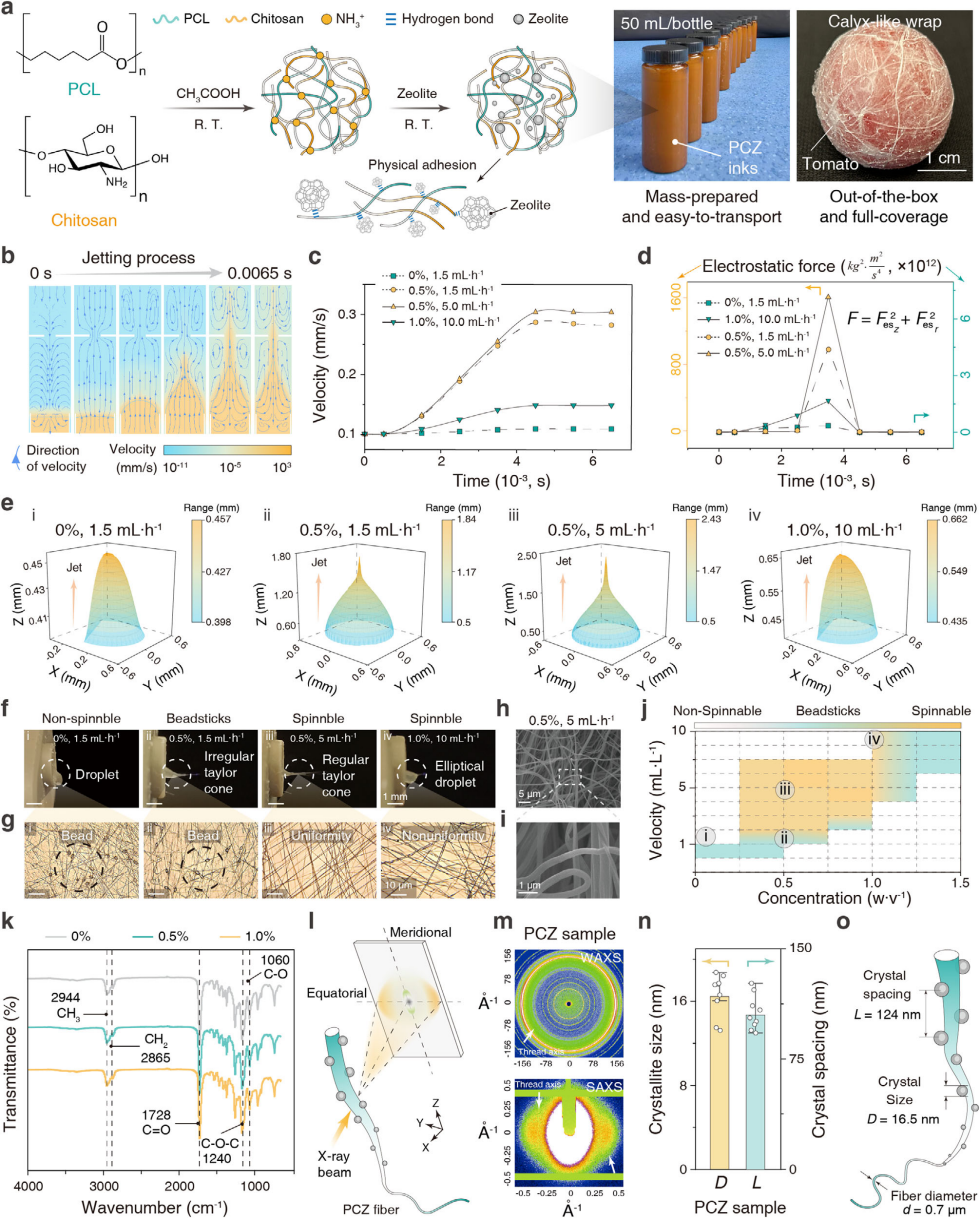
Optimization and multi‐scale structural characteristics of PCZ wrap. (a) Precursor ink prepared by combining PCL, chitosan, and zeolite via hydrogen bonding and electrostatic forces at room temperature. The precursor ink was prepared by compositing PCL and chitosan with zeolite. (b) Multi‐physics finite element simulation of jetting velocity distribution for the optimal precursor ink formulation over time. (c) Average velocity vs. jetting time for different ink formulations. (d) Average electrostatic force vs. jetting time for different ink formulations. (e) Morphology distribution for different precursor ink formulations at 0.0065 s. (f, g) Taylor cone formation (f) and corresponding optical microscopy images of jetted fibers (g). (h, i) SEM images of jetting fibers. (j) Jetting feasibility phase diagram for different precursor inks (The ink can be printed into fiber structures with high fidelity in the parameter range highlighted in yellow. When the jetting velocity and ink concentration are below the threshold value, the ink cannot flow out of the nozzle; on the contrary, when the jetting velocity and ink concentration are far above the critical value, dramatic die swelling occurs, leading to low‐resolution jetting.) Similar observations are shown in (f,g). (k) Fourier transform infrared spectroscopy (FT‐IR) spectra of PCL/CS, PCL/CS/Zeolite (0.5 wt.%), and PCL/CS/Zeolite (1 wt.%), with characteristic peaks. (l) Schematic of the SAXS/WAXS setup for analyzing PCZ fiber micro‐structures. (m) 2D WAXS/SAXS patterns of PCZ fibers. (m) WAXS and SAXS 2D‐scattering patterns of PCZ fibers. (n) Distribution of crystal size and crystal spacing of zeolite particles within the PCZ fibers. (o) Summary of zeolite crystal parameters and PCZ fiber diameter.

To preliminarily investigate the electro‐jetting parameters, the process was simulated using the electrohydrodynamic module in COMSOL software, employing coupled multi‐physical field modeling based on the Taylor and Melcher model [22]. A simplified two‐dimensional axisymmetric model was invoked with the influence factors of gravity, viscous force, surface tension, and electric field force. The ink was jetted at a constant flow rate with voltage applied to generate a Taylor cone. Solvent evaporation within the high‐voltage electric field led to the production of fibers. At a potential difference of 15 kV, we successfully simulated the transition from Taylor cone to stable fiber stream (Figure 2b and Figure S4a). Initially, the liquid formed a hemispherical surface, which gradually elongated into a conical jet over time. Then, this jetting began depositing a layered fibrous network upon the substrate due to inertia caused by its high‐speed jetting. Excessive zeolite concentrations may result in agglomeration within the fiber network, indicating potential clogging in the jetting head. Among the different ink formulations, the ink with 0.5 wt.% zeolite exhibited a higher effective force under the electric field (Figure 2c and Figure S4b), resulting in stable, continuous, and uniform jetting streams.

We conducted further analysis on the impact of electrostatic forces during the jetting process (Figure 2d and Video S1). The ink with 0.5 wt.% zeolite (at 5 mL·h^−1^ flow rate) exhibited the highest electrostatic force within the Taylor cone region. The electrostatic force peaked at 0.0035 s and gradually decreased as the Taylor cone elongated. Analysis at 0.0065 s showed a higher material concentration at the Taylor cone tip for this ink, indicating its superior stability in forming the cone. Overall, this post‐optimized ink formula and jetting parameters exhibited excellent stability and efficiency throughout the entire jetting process.

To verify the optimized jetting parameters, we conducted a dynamic analysis under multi‐physics coupling simulations during fiber formation (Figure 2e). The formation of fibers at varying ink concentrations and flow rates was also captured using a high‐speed camera (Figure 2f), and fiber morphology was observed under optical microscopy (Figure 2g). Fiber distribution was evaluated using SEM (Figure S4c). It showcased defect‐free fibrous network structures at optimized jetting parameters (Figure 2h), resembling the vein structures of the calyx in *Physalis peruviana*. Figure 2i and Figure S4d,e directly revealed the actual spatial distribution and dispersion of zeolite within PCZ fibers. We summarized the law of jetting velocity and zeolite concentration on the manufacture of PCZ wrap, which provided certain guidance for the subsequent scale‐up production (Figure 2j).

### 3.2 Structure and Performance Characteristics of PCZ Wrap

The chemical interactions of PCZ wrap with varying zeolite concentrations were analyzed using FT‐IR. FT‐IR showed a blue shift in the C═O (∼1720 cm^−1^) and C─H (∼2940 cm^−1^) peaks of the sample without zeolite, suggesting enhanced bonding interactions due to zeolite. In CS, minor shifts in the C═O (amide I band, ∼1650 cm^−1^) and N─H bending vibrations (amide II band, ∼1550 cm^−1^) were also observed (Figure 2k). The observed shifts suggest hydrogen bonding between zeolite and functional groups, potentially impacting fibrous structure formation, permeability, and gas molecule interactions. Simultaneously, it was observed that as the zeolite content increased from 0% to 1.0%, the peak center of the C═O stretching vibration shifted from 1728.5 to 1726.9 cm^−^^1^, showing a decrease of approximately 1.6 cm^−^^1^ in wavenumber. This red shift phenomenon indicates enhanced intermolecular interactions as the zeolite content increases. As shown in differential scanning calorimetry analysis (Figure S4f), a certain amount of zeolite as fillers can disrupt the alignment of matrix molecule chains and thus reduce the melting temperature (*T*_m_). Moreover, higher zeolite concentrations promoted filler‐polymer interactions, leading to increased crystalline regions and an increase in *T*_m_. Thermogravimetric analysis (TGA) was employed to quantify inorganic content (Figure S4g). As shown in Figure S4g, TGA revealed weight loss in three stages: removal of moisture below 100°C, decomposition of polymers at ∼100°C–400°C, and stabilization of inorganic components (zeolite) above 400°C.

To further investigate the features of PCZ fiber, wide‐angle X‐ray scattering (WAXS) and small‐angle X‐ray scattering (SAXS) measurements were employed (Figure 2l,m and Figure S4h). The results obtained from WAXS demonstrated a uniform dispersion of zeolite particles within the PCZ fiber, exhibiting consistent crystallite size (*D* = 16.5 nm) and interplanar spacing (*L* = 124 nm) (Figure 2n,o and Figure S4i). Moreover, analysis of SAXS intensity profiles revealed a noticeable peak shift in the PCZ fiber compared to the control (Figure S4j), suggesting alterations in long‐range order and nanoscale structural organization caused by zeolite incorporation. Also, the average diameter of PCZ fiber was obtained (*d* = 700 nm), which was almost consistent with the results in Figure 2h,i. These findings imply that PCZ fiber exhibits uniform yet multi‐scale features.

To evaluate the material's robustness for practical food packaging applications, where mechanical strength and flexibility are crucial for handling and transport and often pose a challenge, tensile stress–strain curves were obtained, demonstrating that PCZ wrap exhibited eminent ductility, elongating up to 85% before fracturing (Figure 3a). Moreover, it displayed a tensile strength of 0.47 ± 0.11 MPa and a toughness of 0.26 ± 0.05 MJ·m^−3^ (Figure 3b), indicating it may satisfy the mechanical properties requirements of a general wrap. Simultaneously, the tensile strength of the PCZ wrap remained statistically stable across all testing time points (Day 0, 7, and 14), indicating that no significant hydrolytic degradation occurred despite the use of an acetic acid‐containing solvent during preparation, which demonstrates excellent mechanical stability (Figure S5). Puncture tests (Figure 3c and Video S2) revealed that the PCZ wrap can withstand forces up to 2.6 N at 11 mm displacement, with a maximum puncture force of 2.14 ± 0.231 N and a puncture energy of 9.02 ± 1.23 MJ (Figure 3d). These results suggest potential tolerance to extreme load scenarios during transportation. Confocal microscopy was employed to examine the post‐puncture micro‐morphology. Plastic deformation and regular perforation were observed (Figure S6a–c), proving that the PCZ wrap has a stubborn network integrity. These remarkable mechanical performances may be attributed to the interaction between components and the resistance of semi‐crystalline or inorganic regions to crack prolongation. To evaluate stability under different storage conditions, we conducted mechanical stability tests in two common representative environments (Env‐1: 25°C, 50% RH; Env‐2: 4°C, 75% RH). Tensile strength and toughness were measured after 0, 3, and 7 days of storage (Figure S6d). No significant changes in mechanical properties were observed under either temperature or humidity condition, indicating that the PCZ wrap maintains good mechanical integrity and structural stability when exposed to moderate environmental fluctuations.

**FIGURE 3:**
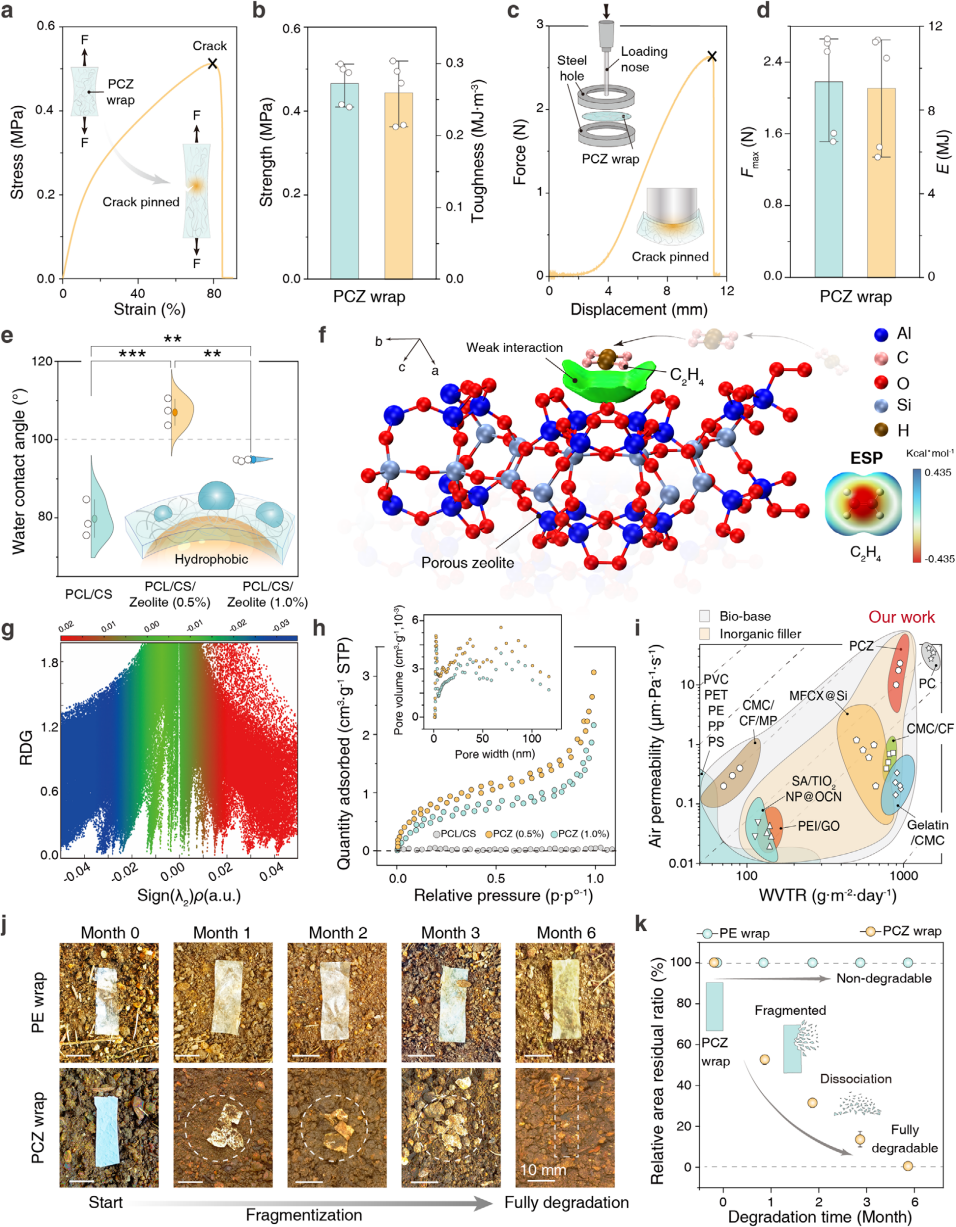
Mechanical properties, air/moisture permeability, ethylene adsorption properties, and biodegradability characteristics of PCZ wrap. (a,b), Mechanical properties of PCZ wrap. Stress–strain curves (a) and tensile mechanical properties summary of PCZ wrap (b). (c,d**),** Puncture tests with force–displacement curves and testing schematic diagram (c), puncture force and fracture energy (d). (e) Contact angle measurements of PCL/CS, PCL/CS/Zeolite (0.5 wt.%), and PCL/CS/Zeolite (1.0 wt.%). (f) Molecular simulation snapshot of ethylene (C_2_H_4_) adsorbed in a porous zeolite channel. The green isosurface highlights the weak interaction region between C_2_H_4_ and the zeolite framework (dominated by *van der Waals* contacts). Inset: electrostatic potential (ESP) mapped on C_2_H_4_ (kcal·mol^−1^). Atom colors: Al (blue), Si (gray), O (red), C (pink), H (brown). (g, h) RDG vs sign(*λ*_2_)ρ plot from IGM/IGMH analysis for the ethylene–zeolite system. The color scale indicates interaction nature: blue (negative) attractive, green (near zero) *van der Waals*, and red (positive) steric repulsion (g), the amount of ethylene adsorption–desorption, and pore size distribution at 77 K (inset) (h). (i) Comparison of PCZ wrap with other packaging wraps in terms of water vapor transmission rate (WVTR) and air permeability. Data are shown in Supplementary Information refs. 8–12. (j, k) Degradation performance of PCZ wrap compared to PE wraps (commercial food wraps from local shop). Degradation progression over 6 months, showing complete breakdown of PCZ wrap while PE wrap remains intact from the appearance of the observation (j). Degradation rate over 6 months, indicating a significantly decreased relative area residual ratio for PCZ wrap and even a 100% degradation at 6 months (k). Data in (b) and (d) are mean ± SD, *n* = 5, and data in (**e**) and (k) are mean ± SD, *n* = 5.

Featuring calyx‐vein‐like layers, interconnected channels at macro, micro, and submicrometer scales were designed to mimic the vein structures of the calyx. In addition to a compact network structure, the introduction of zeolite may reduce wettability and impart hydrophobic as well as air‐permeable properties similar to those found in a calyx. The hydrophobicity was confirmed through contact angle tests, which showed significantly higher contact angles for wrap loaded with 0.5 wt.% zeolite compared to those samples with l wt.% zeolite (109.7° and 109.8° versus 98.0° and 98.01°) (Figure 3e and Figure S6e). Quantitative assessment via TGA tests further supported this hydrophobic character, revealing minimal water uptake with less than 1% weight gain observed over a 7‐day period (Figure S3e). It is attributed to the hydrophobic nature of zeolite and multi‐scale network structures. As zeolite's concentration increases, the network structure loses its integrity, resulting in a significant decrease in hydrophobicity.

The detrimental effects of ethylene on fruits, vegetables, and ornamental plants are estimated to result in product losses ranging from 10% to 80% [22, 23]. Therefore, removal of ethylene is extremely crucial for prolonging the shelf life of perishable foods from farm to table [24, 25]. Here, we elucidated the ethylene adsorption mechanism of the zeolite backbone at the molecular level. The simulation results (Figure 3f,g and Figure S6f,g) showed that ethylene underwent physical adsorption within the ordered microporous channels of *β*‐type zeolite [26, 27]. The zeolite's high surface area and precise pore geometry provided abundant adsorption sites, while the synergistic effect of *van der Waals* forces and weak electrostatic fields enabled ethylene molecules to achieve stable and reversible adsorption without forming covalent bonds. This adsorption mode reflected the non‐reactive gas capture characteristics of the zeolite framework, which ensured an efficient adsorption–desorption dynamic suitable for microenvironment regulation. Further molecular simulation results (Figure 3f) confirmed that ethylene (C_2_H_4_) could enter and stably reside within the pores of *β*‐type zeolite. The ESP distribution of ethylene molecules was relatively uniform, and the visualization of weak interaction regions in the figure suggested that the adsorption between ethylene and the zeolite framework occurred primarily through non‐covalent interactions. To further quantitatively analyze the adsorption process, we calculated the adsorption energy of ethylene on *β*‐type zeolite using DFT (*E*_ads_ = −25.4 kJ·mol^−^^1^), which indicated that the adsorption process was exothermic and dominated by weak interactions. The RDG–sign(λ_2_)ρ scatter plot obtained from IGM/IGMH analysis (Figure 3g) showed that the primary interaction between ethylene and zeolite was dominated by *van der Waals* forces, with weaker contributions from strong attraction and repulsion, exhibiting typical physical adsorption characteristics [28]. Interestingly, by employing physical adsorption principles, we also monitored changes in ethylene adsorption on the PCZ wrap over time (Figure S7). We observed that the dry PCZ wrap exhibited infinite resistance (*R*_∞_). As exposure time increased, the amount of ethylene adsorbed onto the PCZ wrap grew. The interaction between adsorbed ethylene molecules and the composite matrix altered charge transport pathways, leading to a gradual decrease in measured resistance. After 120 minutes, adsorption reached equilibrium. This discrepancy arises from the larger pore sizes within the mesopores and weaker interactions between ethylene molecules and pore walls. We further investigated the effectiveness of removing ethylene from PCZ wrap through adsorption–desorption experiments by Brunauer–Emmett–Teller (BET) [29] (Figure 3h). First, the porous structure of zeolite was confirmed, and the corresponding pore volumes were measured as 0.0045 and 0.0032 cm^3^·g^−1^, respectively. Moreover, the blank groups without zeolite are almost impossible to absorb ethylene, while with an increase in zeolite, there was a corresponding increase in ethylene adsorption capacity. The PCL/CS/Zeolite (1 wt.%) achieved a maximum adsorption of 3.06 cm^3^·g^−1^, while the PCL/CS/Zeolite (0.5 wt.%) exhibited a maximum adsorption of 2.16 cm^3^·g^−1^. This adsorption quantity is enough to remove the ethylene released by the food every day and timely discharge it to avoid food decay.

The presence of various gaseous molecules (e.g., H_2_O, O_2_, CO_2_, etc.) plays a crucial role in the decay mechanism of fruits and vegetables as they regulate plant physiological metabolism [30]. While the oxygen barrier property is important for suppressing microbial growth and regulating respiration, the respiration of fresh fruits and vegetables needs to be dynamically matched with the permeability of packaging materials [31]. Comparing the air permeability and WVTR of PCZ wrap with those of reported and commercial food packagings (Figure 3i), we found that PCZ wrap significantly outperformed (∼1500 g·m^−2^·day versus ∼20 µm·Pa^−1^·s^−1^). This superiority can be attributed to differences in bio‐inspired structures between PCL and conventional wraps. The criss‐crossing multi‐scale structural design of the PCZ wrap effectively reduces the area hindering gas flow, meanwhile, reserving ideal gas permeability and offering an optimal packaging solution for food fresh‐keeping. It is important to note that while general air permeability was assessed (including oxygen transmission), our primary strategy for extending freshness in high‐respiring produce is focused on the active scavenging of ethylene, a key factor in senescence.

Alarmingly conventional cling films, primarily composed of non‐degradable PE, significantly contribute to the approximately 130 million tons of global plastic waste annually [32]. However, even if higher recycling rates are achieved, the life cycle of those non‐degradable plastics may release hazardous endocrine disruptors as well as micro‐ and nano‐plastics into the environment and food chain. This accumulation poses increasingly irreparable risks for chronic diseases and ecological degradation [33]. In biodegradability assessment (Figures 3j,k and S8a), PCZ wrap demonstrated substantial yet complete degradation, with its degradation rate reaching approximately 57% in 1 month and achieving complete degradation within 6 months. In contrast, commercial PE wrap remained largely intact even after 6 months. Quantitative analysis revealed a rapid decrease in the residual area of PCZ wrap, highlighting superior biodegradability compared to minimal changes in PE wraps.

### 3.3 Biological Assessment

The infection and microbial spoilage of foods can be caused by several well‐characterized microorganisms, including both Gram‐positive and Gram‐negative bacteria, as well as fungal agents. These microorganisms can contaminate foods at any stage of the farm‐to‐table supply chain [34]. Therefore, antimicrobial food wrap effectively solves this problem and becomes an effective solution to enhance food safety and extend shelf life [35]. Antimicrobial activity assessments confirmed that PCZ wrap exhibits significant activity against *E. coli* and *S. aureus* (*p* < 0.05) (Figure 4a–d), with no significant inhibitory zone observed around the pure PCL film. The antimicrobial superiority of the wrap can be attributed to the CS [36, 37]. These results indicated that pure PCL wrap exhibits negligible antimicrobial activity, whereas the PCZ wrap demonstrates considerable efficacy against these commonly encountered food strains. Considering the versatility of PCZ wrap in more application scenarios (particularly for pre‐harvest protection of fruits and vegetables), we conducted antiviral tests to evaluate its resistance against tobacco mosaic virus (TMV, RNA virus) [38, 39] (Figure 4e). A significant reduction in active virus particles was observed under transmission electron microscope (TEM) in virus solutions *co*‐cultured with an extract of PCZ wrap for 12 h (Figure 4f). Furthermore, it demonstrated that the application of PCZ wrap significantly mitigated TMV accumulation in the leaves compared to the blank group directly inoculated with TMV (Figure 4g).

**FIGURE 4:**
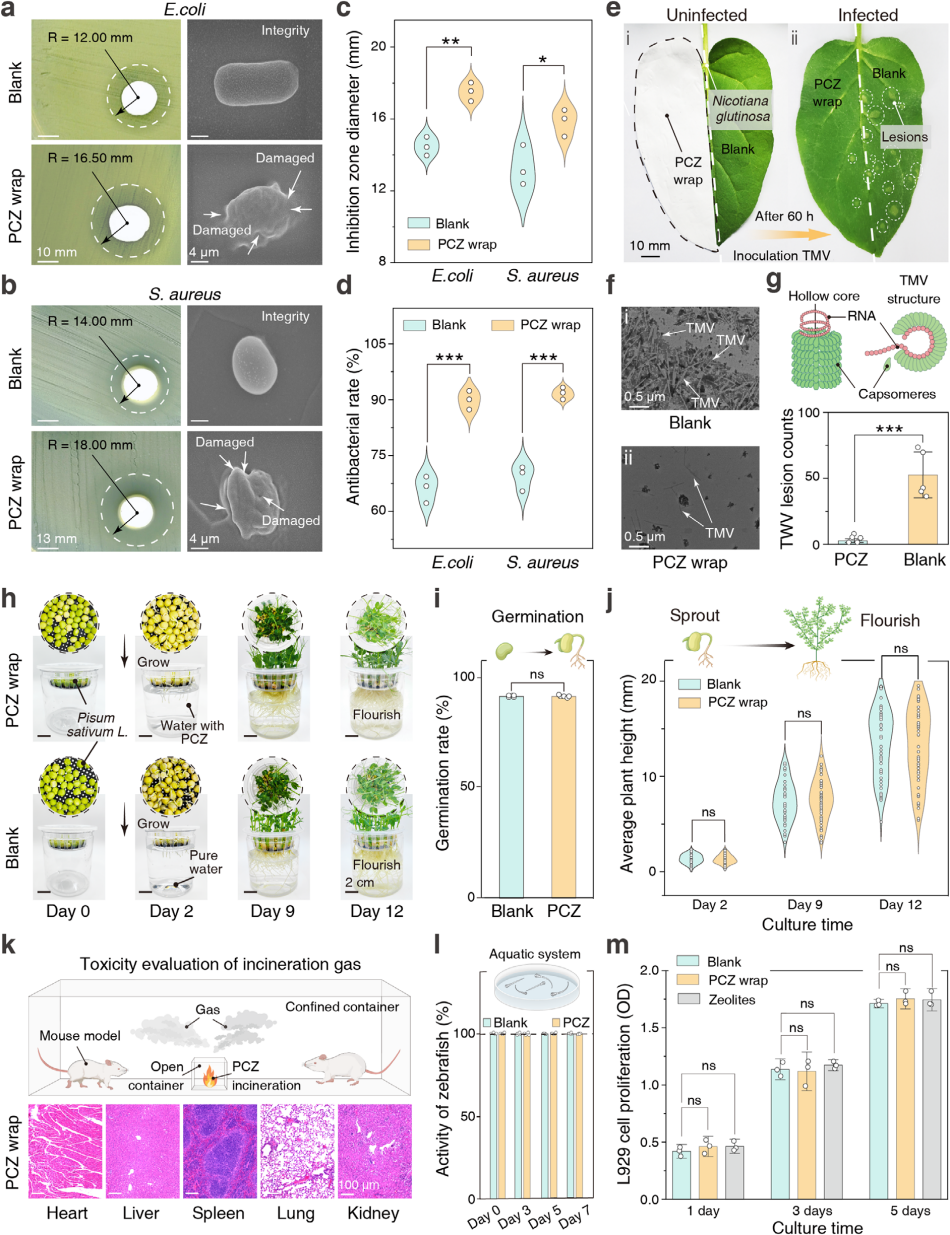
Biological evaluations of PCZ wrap. (a–d) Inhibition zones of *E. coli* and *S. aureus* were examined, the dimensions of the inhibition zones, *n =* 3. (c) Were measured, and the antibacterial rate, *n =* 3. (d) of both was evaluated through morphological observation of the colonies through SEM. (e) Images of TMV infection of *Yerba mate* tobacco leaves (left: covered with PCZ wrap, right: without PCZ wrap). (f) TEM observation of TMV. (g) Comparison of virus spots on the protected side and unprotected side of leaves infected with TMV. The structure of TMV is depicted above. (h–j) Effect of PCZ wrap on seed germination and growth, and schematic representations of the stages from seed germination to growth. (k) Effect of PCZ wrap incineration gas on the respiration of mice. Histological observation (H&E staining) of the mouse heart, liver, spleen, lung, and kidney. Schematic illustration shows the bio‐toxicity evaluation method using mice as mammal models, *n =* 6. (l) Evaluation of fish viability. After a 7‐day culture, zebrafish co‐cultured with PCZ wrap maintained a high vitality, indicating the PCZ wrap is biosafe and nontoxic, *n =* 7. (m) CCK‐8 assay of L929 cell viability after incubation with blank medium, PCZ wrap extracts, and zeolite extracts for 1, 3, and 5 days, *n =* 3. Data in (c), (d), (g), (i), (j), (l), and (m) are mean ± SD.

To assess the bio‐safety of PCZ wrap as a food packaging material, we conducted a series of international standard tests (FDA 21 CFR 170–199) for the food industry. In a phytogenic growth experiment involving hydroponically cultivated bean sprout seeds, the seeds were incubated for 12 days at room temperature in purified water containing 1 mg·mL^−1^ of PCZ wrap. The results demonstrated almost no impact on sprout growth or coloration, with a germination rate of 94% and no noticeable difference in plant height (Figure 4h–j). These findings strongly suggested that PCZ wrap does not exert any adverse effects on the plant ecosystem. Furthermore, the impact of immediate contact and post‐treatment of food wraps on the animal ecosystem was of greatest concern. Incineration is one of the most commonly used post‐treatments for waste food packaging. Here, histological analysis displayed no significant differences in tissue morphology compared to the blank group. Moreover, the response of aquatic organisms is crucial for evaluating water ecosystem safety [38]. Therefore, we performed aquatic toxicology tests by culturing zebrafish in water containing 10 mg of PCZ wrap for 7 consecutive days. The zebrafish exhibited good survival status (Figure 4l and Video S3). HE staining of zebrafish liver and kidney tissues was also examined after 7 days (Figure S9a,b). Compared to the blank control group, no inflammatory lesions were observed in the PCZ wrap group, indicating that PCZ wrap exhibits no toxic effects on aquatic ecosystems. As shown in Figure 4m, no significant reduction in cell viability was observed at any exposure time point, indicating that the zeolite‐containing extracts exhibit no detectable cytotoxicity under the tested conditions. These results provided experimental evidence that the incorporation of zeolite does not introduce observable biological safety risks related to cytotoxicity. An incineration gas inhalation test was employed to evaluate the air ecosystem safety of PCZ wrap (Figure 4k and Figure S8b). It revealed that gases released from the combustion of PCZ wrap did not negatively affect the physiological condition of mice (Video S4). Collectively, these results validated the outstanding biosafety of PCZ wrap, thereby supporting its foreground in the food industry.

### 3.4 Practical Application of PCZ Wrap

The portability of HED and the superior fresh‐keeping performance of PCZ wrap offered an unprecedented and highly efficient solution for food preservation (Figure 5a and Video S5). To comprehensively evaluate its efficacy, we selected representative fruits with differing physiological characteristics based on the core classification standard of fruit ripening's respiratory metabolic pattern [40, 41]. Specifically, we chose cherries (Figure 5) as a typical non‐climacteric fruit and tomatoes (Figure S10a,b) as a typical climacteric fruit and concurrently supplemented our study with two additional typical climacteric fruits, namely bananas and mangoes (Figure S10c,d). These commercially valuable and perishable fruits were utilized as food models and investigated through a series of in‐depth preservation tests. At the retail stage, variations in fruit appearance (color, chromaticity, and surface mildew), fruit softening, and dehydration are major symptoms of quality loss.

**FIGURE 5:**
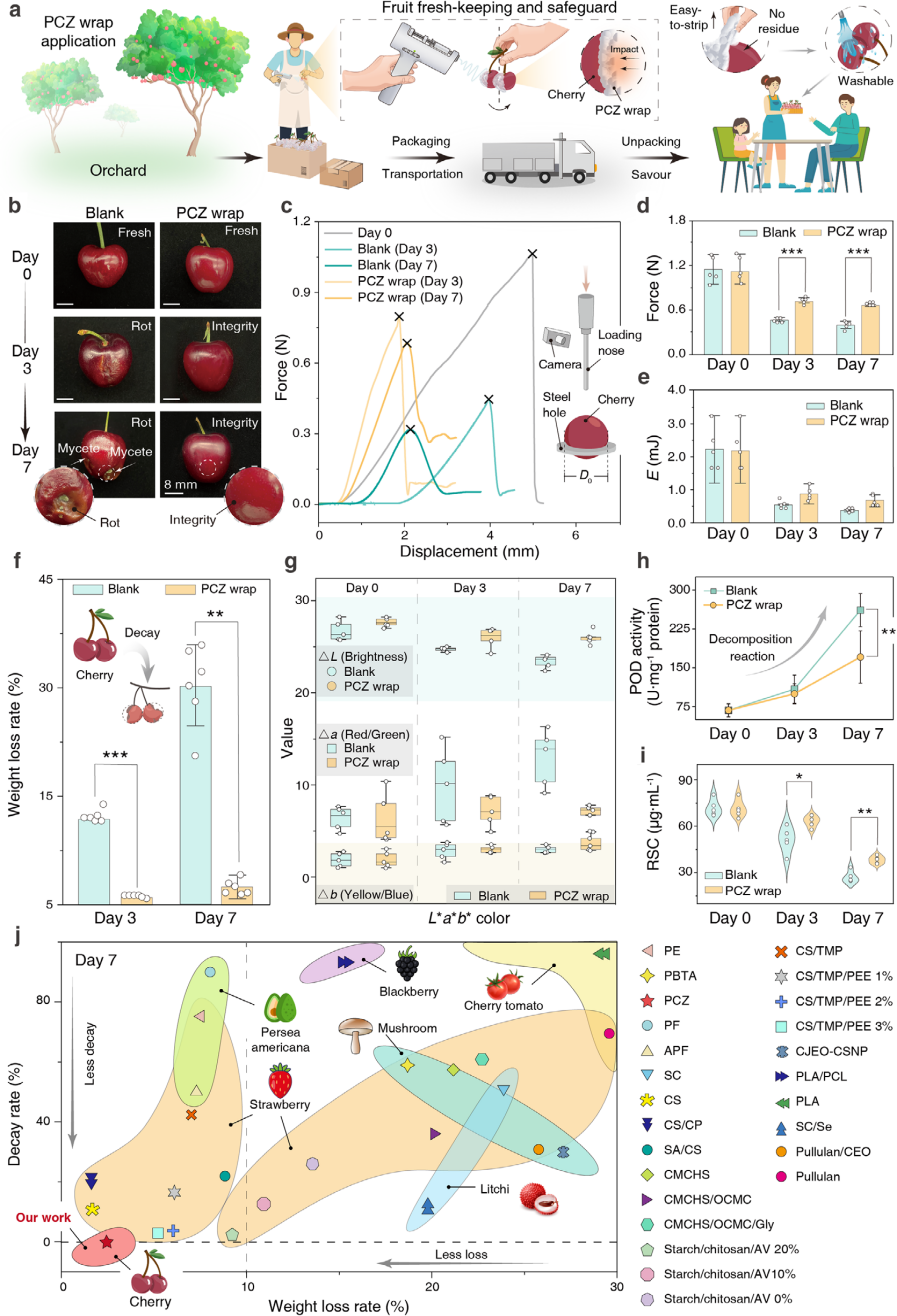
Effect of PCZ wrap on food quality. (a) Schematic diagram of PCZ wrap practical application. PCZ wrap provides an efficient solution for food fresh‐keeping from farm to customer, with long‐term effect, easy‐to‐strip, residue‐free, and washable features, ensuring high quality and convenience from fruit harvesting to the consumer. (b) Appearance of cherries with and without PCZ wrap stored at room temperature for 0, 3, and 7 days. The cherries without PCZ wrap (blank group) show mold and wrinkling. (c–e) Firmness tests of cherries with and without PCZ wrap stored at room temperature for 0, 3, and 7 days. Puncture test was employed to test the firmness of the fruit, as shown in the embedded schematic diagram. Force–displacement curves (c), puncture force (d), and energy (e) are shown. (f–i) Analysis of weight loss (f), color changes (g), POD enzyme activity (h), and reducing sugar content (RSC) (i) of cherries with and without PCZ wrap after storage at room temperature for 0, 3, and 7 days. (j) Comparative evaluation of different fresh‐keeping wraps applied to various fruit models, assessing both decay rate and weight loss rate over a one‐week storage period. Data are shown in Supplementary Information refs. 25–35. Data in (d), (**e**), (f), (g), (h), and (i) are means ± SD, *n* = 5.

Cherries treated with PCZ wrap and stored at 25°C for 7 days exhibited excellent resistance to mold, as evidenced by their intact appearance, unlike the blank group, where mold growth was clearly observed (Figures 5b and S10e,f). Similar results were also obtained with tomatoes (Figure S10a,b and Video S6). Building on this, to further validate the efficacy of our strategy on climacteric fruits, we additionally conducted preservation experiments on bananas and mangoes, comparing them with common PE film (Figure S12). Usually, firmness loss is an intuitive way to determine whether the fruit is fresh. In terms of firmness tests, cherries treated with PCZ wrap exhibited the highest firmness (∼0.85 N) on day 3 and maintained a firmness of 0.7 N after 7 days, which was significantly higher than that of the blank group (Figures 5c–e, S10g, and Video S7). Moreover, maximum puncture force and energy were significantly higher in tomatoes wrapped with PCZ compared to those untreated (Figure 5c,d), demonstrating the preponderance of PCZ wrap in preserving food freshness.

Weight loss assessment of cherries further substantiated the superior efficacy of PCZ wrap. Following a 3‐day storage at 25°C, the blank group exhibited a weight loss of 12.4%, while the PCZ wrap group demonstrated only 5.2%, representing a more than twofold reduction. After 7 days, weight loss reached 31.1% in the blank group compared to only 8% in the PCZ wrap group (Figure 5f), indicating an almost fourfold improvement in fresh‐keeping (Figure 5f). Similar trends were observed in tomatoes, where the blank group exhibited approximately four times the weight loss of the PCZ group on day 3 and eight times on day 7 (Figure S10h). Furthermore, apples and pears wrapped with PCZ wrap maintained a stable appearance and exhibited lower weight loss during storage (Figure S11a,b).

Chromaticity variations, calculated based on the CIELAB color space, are important indicators of fruit freshness [42]. A decrease in L* value reflects diminished gloss and surface brightness, while an increase in a* value signifies a transition toward redness during ripening [43]. Color analysis by Day 7 (Figure 5g and Figure S12a–f) revealed reduced brightness (decreased L*) and a significant increase in redness (a* value, *p* < 0.05) for the blank group, indicative of overripeness or decay. Furthermore, there was a slight decrease in the b* value, resulting in a bluish tint and darker appearance. In contrast, the PCZ wrap group maintained stable L* and a* values with minimal change observed in b*, signifying the reduction of spoilage and preservation of its natural hue. These findings collectively underscore that PCZ wrap significantly preserves fruit quality by inhibiting mold growth, maintaining firmness, effectively reducing weight loss, and preserving natural chromaticity.

Under identical storage conditions, four representative fruits (bananas, mangoes, tomatoes, and cherries) were packaged using commercial PLA film and continuously monitored for 7 days. The quantitative evaluation metrics included appearance, weight loss, and color parameters (Figure S13a–d). As shown, all PLA film fruits exhibited noticeable surface deterioration and color darkening by day 7, indicating limited preservation capability. In contrast, under the same conditions, the PCZ wrap demonstrated significantly superior appearance stability and slower quality deterioration, offering distinct advantages. Furthermore, we employed non‐degradable materials (aluminum foil) to package bananas and mangoes, continuously monitoring them for 7 days under the same storage conditions as above. Visual inspection revealed (Figure S12 and Table S2) that bananas and mangoes packaged with aluminum foil exhibited significant surface deterioration and color darkening by day 7. In contrast, the PCZ wrap demonstrated significantly superior appearance and storage stability. Among them, after storing bananas with the PCZ wrap for 7 days, the peel was sampled and examined for surface residues using SEM. As shown in the results (Figure S13e,f), no residues were detected on the peel after storage.

Ethylene accelerates fruit ripening by promoting amylase, enhancing RNA and protein synthesis, and increasing cell membrane permeability [44]. During ripening, POD, a critical oxidoreductase, regulates physiological changes [45]. In this study, we investigated POD activity and RSC in cherries and tomatoes after 7 days of storage at 25°C. The results demonstrated that the blank group exhibited significantly higher POD activity compared to the PCZ wrap group (Figure 5h and Figure S10i), indicating PCZ wrap effectively slowed down POD activity to delay ripening by removing ethylene. Although both groups showed a decrease in RSC (Figure 5i and Figure S10j), the reduction was less pronounced in the PCZ group, suggesting its inhibition of starch and carbohydrate hydrolysis. We further summarized the decay rate and weight loss rate of various fresh‐keeping wraps after one week of storage (Figure 5j). The results indicated that the PCZ wrap performed best on cherries after one week of storage, showing no decay and a weight loss of only 2.49%, which highlights its superior fresh‐keeping capabilities.

Distinct from other reported approaches, we have successfully applied for a patent for this fresh‐keeping strategy as an integrated product and have initiated its implementation in practical settings. In practical applications (Figure S14a–c), the portability and user‐friendliness of the HED enable farmers to implement fresh‐keeping measures swiftly on‐site, thereby protecting fruits from physical damage, microbial infestation, and immediate water loss after harvesting and minimizing economic losses due to quality degeneration. For consumers, PCZ wrap effectively sustains fruit freshness during transportation and storage, prolonging their shelf life, resulting in preserved nutritional value and flavor, ultimately enhancing market appeal and consumer confidence.

To evaluate the thickness uniformity of the PCZ wrap preparation, we quantitatively analyzed the thickness of the PCZ coatings deposited on the surfaces of four representative fruits (tomatoes, cherries, mangoes, and bananas) using a handheld electrostatic deposition device (Figure S15a,b). The coating thickness exhibited a stable distribution within the 15–20 µm range, with no statistically significant differences observed between operators (adults and children) or fruit types. These results indicate excellent uniformity and ease of operation for the coating process.

### 3.5 Non‐Targeted Metabolomics and Environmental Assessments

Metabolomics research, focusing on the investigation of metabolite changes, plays a crucial role in understanding food fresh‐keeping mechanisms [46]. To explore the metabolic alterations associated with cherry fresh‐keeping under a long‐standing treatment of PCZ wrap, non‐targeted metabolomics analysis was employed. This approach provides a comprehensive overview of all detectable primary and secondary metabolites, enabling the assessment of nutritional preservation and identification of defense‐related metabolites involved in aging and spoilage. Utilizing chromatography‐mass spectrometry (GC/LC‐MS/MS), non‐targeted analysis identified 2725 metabolites in untreated and treated cherries following 7 days of storage (Figure 6a,b and Table S3). Quality analysis confirmed the reliability and representativeness of these data (Figure 6c and Figure S16a).

**FIGURE 6:**
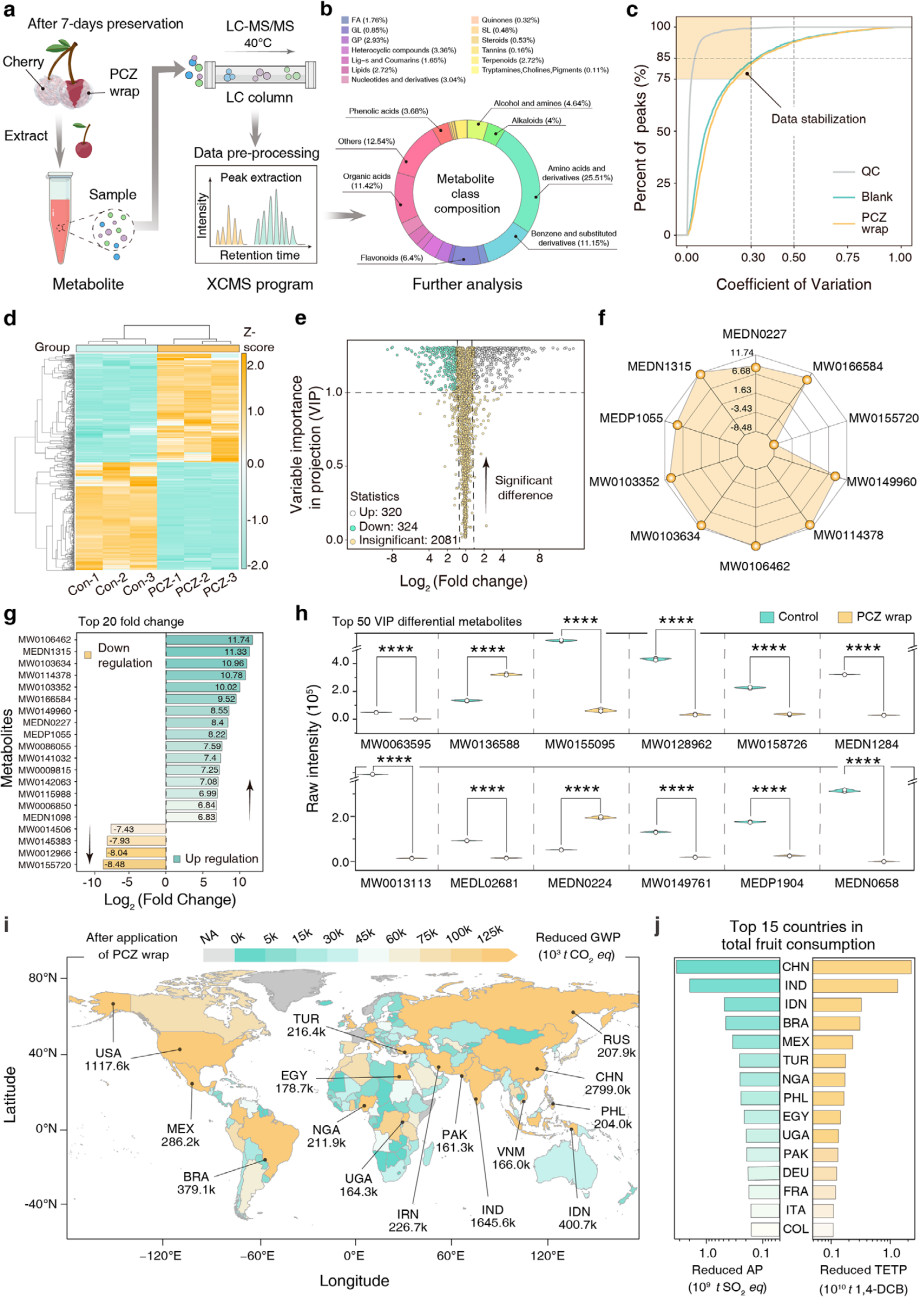
Metabolomics analysis of cherries after fresh‐keeping and simulation assessment of the environmental impact of PCZ wrap. (a,b) Schematic workflow of untargeted metabolomics analysis for cherries preserved with PCZ wrap. (c) Coefficient of variation (CV) plot demonstrating high stability of experimental data. (d) Clustering analysis of metabolites and samples; the left cluster dendrogram represents metabolites, while the top cluster dendrogram represents samples. (e) Volcano plot of differential metabolites. (f) Top 10 metabolites with the highest absolute log2 fold change across different groups. (g) Top 20 metabolites ranked by fold change across groups. (h) Relative intensity of the 12 most significant differential metabolites among the top 50 VIP metabolites. (i) Environmental impact model based on 2021 per capita fruit consumption data is used to evaluate the global application of PCZ wrap. Compared to conventional food fresh‐keeping wraps, the usage of PCZ wrap significantly reduces carbon emissions in various countries, notably for China, India, and the USA, which exhibited substantial improvements in environmental impact and carbon reduction effects (15 countries in total). (j) Acidification potential (AP) and terrestrial ecotoxicity potential (TETP) reductions for the 15 countries with the highest total fruit consumption.

A supervised orthogonal partial least squares‐discriminant analysis (OPLS‐DA) model was constructed to investigate metabolic disparities between treated and untreated cherries, exhibiting excellent fit metrics (*R* ^2^ *X* > 0.5, *R* ^2^ *Y*, *Q* ^2^ close to 1; Table S4). Differential metabolites were identified using variable importance in projection (VIP > 1) and fold change (|Log_2_FC| ≥ 1.0) criteria for subsequent two‐group analysis. Significant metabolic differences were highlighted by the OPLS‐DA S‐plot (Figure S16b), which also identified key metabolites contributing to group separation. The dynamic distribution of fold changes (log_2_FC) (Figure S16c) further emphasized specific upregulated and downregulated metabolites after treatment.

Hierarchical clustering analysis and volcano plot analysis revealed distinct clustering patterns among metabolites, with 320 upregulated and 324 downregulated metabolites, emphasizing substantial variations in abundance between groups (Figure 6d,e). From the quantitative analysis, we identified the top ten metabolites with the highest absolute log_2_FC values, which encompassed five amino acid derivatives, one lactone, one sugar molecule, two organic acids, and one amine compound (Figure 6f). Fold change analysis of the top 20 metabolites (Figure 6g) showed significant variation, while VIP score analysis in the OPLS‐DA model identified the top twenty metabolites most responsible for group separation (Figure S16d). This enhanced our understanding of the metabolic impact of PCZ wrap treatment. Furthermore, the correlation and *Z*‐score analyses conducted on differential metabolites (Figure S16e,f) further illustrated the relationships and distribution patterns among the top 50 metabolites with the highest VIP scores, revealing notable associations and discrepancies among these crucial molecules. These findings ultimately reflect the effectiveness of PCZ wrap in modulating essential metabolic interactions aimed at improving fruit freshness.

Previous studies have demonstrated that fruits undergo significant metabolic changes during ripening and after harvesting, particularly involving the accumulation of amino acids and their derivatives, carbohydrates, alkaloids, and flavonoids. These changes reflect active metabolic processes occurring at this stage [47, 48]. Primary metabolites encompass amino acids, carbohydrates, and fatty acids, whereas secondary metabolites (e.g., alkaloids and flavonoids) possess antimicrobial and antioxidant properties. In the untreated cherry group, noticeable alterations in key metabolites such as amino acids, carbohydrates, alkaloids, and flavonoids were observed. This indicates enhanced metabolic activity, which may contribute to accelerated ripening and quality deterioration (Figure 6h). Conversely, the PCZ wrap group exhibited significantly distinct metabolite profiles, showing notable reductions in certain metabolites (e.g., amino acids, piperidine alkaloids) and noteworthy increases in others (e.g., saccharides). These findings suggest that PCZ wrap facilitates a well‐regulated metabolic state conducive to fruit fresh‐keeping. These discernible differences imply that PCZ wrap effectively modulates metabolic pathways by reducing aging‐related processes and oxidative stress. Such balanced regulation considerably retards spoilage, thereby extending the shelf life of cherries while further validating the advantages of PCZ wrap in maintaining fruit freshness.

Finally, the utilization of the Kyoto Encyclopedia of Genes and Genomes functional annotation and enrichment analysis facilitated the classification, pathway enrichment, and differential abundance analysis of metabolites. This analysis further confirmed significant enrichment in crucial pathways, including carbon fixation, glycerophospholipid metabolism, and glutathione metabolism. These outcomes effectively emphasize the regulatory role of PCZ wrap in modulating cherry metabolism (Figure S16g–i). Consequently, these findings indicate that PCZ wrap proficiently prolongs cherry freshness by modulating metabolites associated with aging and oxidative stress. As a result, this study establishes a fundamental basis for targeted metabolomics to delve deeper into preservation‐related metabolic pathways while identifying key regulatory mechanisms.

The role of PCZ wrap was further confirmed through untargeted metabolomics analysis. Importantly, it not only prolongs the shelf life of fruits but also contributes to sustainable food fresh‐keeping practices. Considering the environmental challenges posed by conventional non‐degradable wraps, it is crucial to assess the environmental impact of PCZ wrap as a sustainable alternative. It should be noted that most currently available food wraps on the market are made from non‐biodegradable plastics, which continue to exert significant pressure on the environment [11]. As of 2018, approximately 4 million tons of non‐biodegradable wraps were produced annually, with a majority ending up as global waste [49]. This surge in plastic production has resulted in increased plastic waste and the release of associated chemicals and by‐products into the environment throughout their lifecycle [50].

Therefore, a cradle‐to‐gate LCA [51] was conducted to quantify the environmental impacts of PCZ wrap and visually compare it against four common plastic films (Figure S17j), where darker shades represent higher environmental impact levels. To comprehensively assess the environmental impact of PCZ wrap in the market, we integrated the demand growth model using fruit consumption data in 2021 and population projections for 2024 to estimate its global environmental impact [52, 53]. Given its biodegradability, we evaluated its effects on GWP, AP, and TETP for large‐scale application in 2024.

Large‐scale application simulations (For standardization and comparative modeling purposes in our work) of PCZ wrap have demonstrated significant reductions in GWP, particularly in China and India, with respective reductions of 2799 × 10^6^ t CO_2_ eq. and 1645.6 × 10^6^ t CO_2_ eq. (Figure 6i). This is equivalent to emissions avoided by approximately 608 × 10^6^ cars for 1 year (each emitting ∼4.6 t CO_2_ annually) or the carbon absorption capacity of nearly 127,227 × 10^6^ mature trees annually (each absorbing ∼22 kg CO_2_ per year), effectively contributing to climate change mitigation [54, 55]. Among the top 15 countries with the highest per capita fruit consumption, China exhibited the most notable decreases in AP and TETP (Figure 6j), achieving reductions up to 3.28 × 10^9^ t SO_2_ eq. and 2.32 × 10^10^ t 1, 4‐DCB eq., respectively. Although these results are based on idealized assumptions that may not fully reflect market complexities, they still indicate the potential sustainability benefits of PCZ wrap in food fresh‐keeping practices. The analysis assumed electricity from an average Chinese grid; electricity consumption during preparation accounted for about 62% of the environmental impact, primarily due to reliance on coal. Transitioning to renewable energy sources could further reduce this impact. Overall, the findings suggest that PCZ wrap offers a more sustainable approach to food fresh‐keeping by mitigating global warming while delivering environmental and economic advantages.

## 4 Discussion

The future packaging economy should be centered on sustainable consumption and production practices, which is one of the important signals for the United Nations Sustainable Development Goal (SDG12) [14]. It contributes to guiding the rational use of resources and production technologies, providing adequate product performance, ensuring sound waste management, and minimizing the environmental impacts. Inspired by the unique structure‐function relationship of the *Physalis peruviana* calyx and driven by sustainability principles, we successfully developed a straightforward, highly efficient, and eco‐friendly food fresh‐keeping strategy. Full‐chain standard evaluations in this work have already confirmed its superior effectiveness, feasibility, and practicability. Economically, it enhances economic sustainability by reducing food waste, transportation, and preservation costs throughout the entire food supply chain and even mitigates waste management costs. Socially, customers can enjoy fresh and healthy foods, effectively improving the residents' quality of life. Environmentally, PCZ wrap decreases the environmental footprint during both production and disposal stages, reduces greenhouse gas emissions, and poses no risk to ecological safety.

In order to speed up this fresh‐keeping strategy in the market, we have carried out downstream cooperation with local agricultural enterprises to conduct ongoing pilot evaluations in a greenhouse orchard with ∼30,000 m^2^ (located in Dapeng New District, Shenzhen city). We have actively received feedback and suggestions from the entire supply chain and generated the product market research reports. According to the previous reports, conceivably, the leading concern for farmers may be the cost. In this strategy, the main cost comes from PCZ ink, which acts as a consumable product, and its mass preparation enables a low cost (< 0.0005 $·cm^−2^), equivalent to ∼0.2 times PE plastic wrap in the current online market (http://www.amazon.com). Certainly, as the production scale is expanded, this cost gap will be further increased. For consumers, the biggest concern remains food biosafety. Although we have obtained comprehensive biosafety support in this work, we are still working with local hospitals to track the long‐term biosafety of the PCZ wrap. So far, the overall market research results are positive for this strategy. Notably, being portable and user‐friendly, the PCZ wrap can be directly utilized by consumers for keeping take‐home foods fresh, thus expanding the market beyond B2B to include B2C.

Certainly, this work presents opportunities for future improvement and exploration. First, while PCZ wrap has demonstrated significant preservation efficacy for both non‐climacteric (e.g., cherries) and climacteric fruits/vegetables (e.g., tomatoes, bananas, and mangoes), its application to preserving peeled fruit flesh warrants further investigation. Furthermore, the potential of PCZ wrap in other food categories such as ready‐to‐eat meals, dairy products, vegetables, and meat necessitates further investigation. Additionally, the preservation of fruit is closely related to the temperature and humidity of the surrounding environment. The implementation environment in this work is located in Shenzhen city, China (subtropical monsoon climate) with latitude (114°East, 22.5°North), diurnal temperature range (28°C–35°C), and humidity range (70%–90%); future work can take global environmental factors and seasonal factors into account. Moreover, this study aims to demonstrate its comprehensive functional advantages for applications such as on‐site or conformal coating rather than maximizing its intrinsic mechanical strength. Therefore, future work can take the mechanical performance of the PCZ wrap into account to unlock more application scenarios. Future research should prioritize the functionality, sustainability, and scalability of PCZ wrap for a wider range of food categories, thereby advancing safety and sustainability within the food industry.

## 5 Conclusions

In conclusion, the design of PCZ wrap seamlessly integrates sustainability throughout the entire supply chain, spanning from production to consumption, aligning with the objectives of the sustainable food industry. It presents a promising solution for developing a new generation of biodegradable fruit coatings with active preservation capabilities, contributing to the establishment of a greener, low‐carbon, and highly efficient food supply chain.

## Author Contributions

Xiangyu Liang and Yue Zhao conceived and designed the experiments. Yue Zhao and Chengbang Lu fabricated all materials. Xiaopeng Bai and Chengbang Lu performed the preparation process simulation experiments and analyses. Xiangyu Liang, Yue Zhao, and Chengbang Lu performed the macroscopic feature imaging and mechanical characterizations measurement. Danlei Sun, Binxin Liang, Yunxiang Zhang, Zeyu Wang, and Hong Zhang performed the structural characterizations measurement. Hong Zhang and Yue Zhao conceived and directed the animal and cellular experiments. Yi Yang, Yuqin Hu, and Yingning He performed the microbial characterizations measurement; Shangyi Wu and Xinjie Wang performed the metabolomics characterizations measurement and analysis; Taolin Sun, Zeyu Wang, Shangyi Wu, and Shengwen Duan performed the food chemical characterizations evaluation. Chengbang Lu and Xiaopeng Bai performed the simulation assessment of the environmental impact. Yang Luo, Yue Zhao, and Chengbang Lu wrote the manuscript. Hong Zhang, Yang Luo, and Xiangyu Liang supervised the entire work. All authors discussed the results and commented on the manuscript.

## Conflicts of Interest

The authors declare no conflicts of interest.

## Supporting information




**Supporting File 1**: exp270196‐sup‐0001‐SuppMat.docx.

**Supporting File 2**: exp270196‐sup‐0002‐Video1.mp4.

**Supporting File 3**: exp270196‐sup‐0003‐Video2.mp4.

**Supporting File 4**: exp270196‐sup‐0004‐Video3.mp4.

**Supporting File 5**: exp270196‐sup‐0005‐Video4.mp4.

**Supporting File 6**: exp270196‐sup‐0006‐Video5.mp4.

**Supporting File 7**: exp270196‐sup‐0007‐Video6.mp4.

**Supporting File 8**: exp270196‐sup‐0008‐Video7.mp4.

## Data Availability

Supplementary information and chemical compound information are available in the online version of the paper. Reprints and permissions information is available online.
