# Supplementary material for: A High‐Efficiency Yet Sustainable Fresh‐Keeping Strategy Inspired by Calyx of Physalis peruviana
Source: Exploration (Beijing). 2026 Jul 29;6(5):20250786. doi: 10.1002/EXP.20250786 (PMC13420316; doi:10.1002/EXP.20250786)
Supplement: Supplementary file 1 [file EXP2-6-20250786-s006.docx]

**Supplementary Information**

**A high-efficiency yet sustainable fresh-keeping strategy inspired by [calyx](https://en.wikipedia.org/wiki/Calyx_(botany)) of *Physalis peruviana***

**S.I. Experimental details**

**Materials and chemicals**

Unless specified, the materials and chemicals used in this work were used without further purification. Polycaprolactone (PCL, average *M*_w_ ~ 80 kDa) was purchased from Mackin (China). Chitosan (CS, from shrimp shells, deacetylated ≥ 75%) was purchased from Sigma-Aldrich (Germany). Formic acid (FA, A.C.S-reagent, 98%) and Acetic acid (AA, A.C.S-reagent, 99.7%) was purchased from Sigma-Aldrich (Germany). Fresh tomatoes (variety: *Sweet 100*, weight: 80-150 g, origin: Italy), fresh cherries (variety: *Santina*, size: 24.6-26 mm, origin: Chile), bananas (variety: *Williams*, weight: 160-180 g, origin: Guangxi, China), mangos (variety: *Tainong No.1*, weight: 120-140 g, origin: Hainan, China), apples (Variety: Red Fuji, Weight: 180-195 g, Origin: Shandong, China), and pears (Variety: Fragrant Pear, Weight: 150-165 g, Origin: Xinjiang, China) were purchased from local Chinese fruit markets (Table S1). Handheld electro-jetting device (HED) was specially designed and manufactured by a cooperation with Qingdao Nuokang Environmental Protection Technology *co.*, Ltd (Qingdao, China). Zeolites (*β*-cage: a large cage, 11.6 *Å*; pores, 4.4 *Å*; length of a side, 6.6 *Å*) were purchased from Sigma-Aldrich (Germany). Commercial cling wrap (Aluminum Foil, PE and PLA) was purchased from Jiangsu Nantong Huitong Plastic Machinery Co., Ltd. (Jiangsu, China) and used as a control. *Physalis peruviana* were obtained by cultivated in our laboratory.

**Design and assembling of handheld electro-jetting device (HED)**

The HED device, operating in cyclic charging mode with constant voltage, was employed to fabricate the PCZ wrap (Figure S1). Specifically, the wireless HED weighs approximately 350 g and dimensions of 20 cm in length and 15 cm in width. Its key features include portability, single-handed operation suitable for one individual, fast-charging capability, 4 h continuous jetting, and a long standby time of 30 days.

**Preparation of** **PCL/CS/****Zeolite (PCZ) inks**

For the preparation of PCZ wrap, a jetting ink containing PCL, chitosan and zeolite was prepared. In brief, the CS (2 *wt.*%) solution was prepared by dissolving 1 g of CS in 50 mL of the FA:AA solvent system (1:2). Then 6 g of PCL particles were added and magnetically stirred at 50 °C until complete dissolution. Afterward, zeolite was added into mixed solutions (PCL and CS) with a different mass ratios of 0%, 0.5% and 1%, respectively, under magnetic stirring at room temperature. The inks were loaded into a plastic syringe (5 mL) and delivered to the spinneret using a syringe pump. PCZ jetting fibers were deposited directly onto the fruit epidermis, and the mass per unit surface area was adjusted by varying the collection time. After simulations and experimental studies for each parameter, the final parameters were set as follows: PCL (12%, w/v), CS (2%, w/v) , and zeolite (0.5%, w/v) , with a ink flow rate of 7.5 ~ 10 mL·min^-1^, and this film was referred to as PCZ wrap.

S. **II. Instrumentation and measurements**

**Molecular weight measurements**

The average molecular weight (*Mw*) was determined using an Alliance GPCV 2000 (Waters Corporation, USA) at 30°C. The mobile phase with hexafluoroisopropanol, was eluted at a flow rate of 1 mL·min^-1^. A calibration curve was plotted using polystyrene standards. Molecular weight was determined by gel permeation chromatography (GPC) [1] .

**Spinnability Simulation**

The Taylor cone simulation was conducted using COMSOL 6.2 software, employing a multiphysics coupling modeling approach. To characterize the changes in the liquid surface morphology, a two-phase flow model was established, with one phase representing the fluid and the other representing air. Given the symmetrical structure of the Taylor cone [2, 3] a two-dimensional axisymmetric model was constructed to reduce computational load. The multiphysics interface of COMSOL was set to the two-phase flow interface, and the moving interface was set to level set. In the electrostatic physics interface, the negative pole was grounded, and the positive pole was connected to the nozzle of the syringe needle and voltage was applied. The needle electrode was set to 15 kV, consistent with the working voltage of the handheld electro-jetting machine. The solution density was set to 1165 kg·m^-3^, consistent with the density of the PCL/CS/Zeolite solution. The relative gas permittivity was set to 1, and the relative liquid permittivity was set to 60 [4]. The COMSOL simulation time ranged from 0 to 0.0065 s.

In the simulation process, in order to calculate the current density distribution inside the liquid, the following *Equation* (1) is used:

$\text{J}_{\text{inwrad}}\text{ =}\text{u}_{\text{r}}\text{×}\text{j}_{\text{r}}\text{+}\text{u}_{\text{r}}\text{×}\text{j}_{\text{∅}}\text{+}\text{u}_{\text{z}}\text{×}\text{j}_{\text{z}}$	(1)

where $\text{u}_{\text{r}}$, $\text{u}_{\text{r}}$ and $\text{u}_{\text{z}}$ are unit vectors of radial, azimuth and axial angles respectively; $\text{j}_{\text{r}}$,${\text{ }\text{j}}_{\text{∅}}$, and $\text{j}_{\text{z}}$are the components of the current density in these directions, respectively.

To describe the charge density on a liquid surface, the following *Equation* (2) is used:

$\text{σ}_{\text{surface}}\text{ =}\text{u}_{\text{r}}\text{×}\text{D}_{\text{r}}\text{+}\text{u}_{\text{∅}}\text{×}\text{D}_{\text{∅}}\text{+}\text{u}_{\text{z}}\text{×}\text{D}_{\text{z}}$	(2)

where $\text{D}_{\text{r}}$, $\text{D}_{\text{∅}}$, and $\text{D}_{\text{z}}$ are the radial, azimuth, and axial components of the electric displacement vector, respectively.

When considering the effect of gravity on the fluid, the following weak form *Equation* (3) is used:

$\text{W}_{\text{gravity}}\text{=2π}\text{r}\left( \text{F}_{\text{g,r}}\text{× test}\text{ }\text{(}\text{u}\text{)+}\text{F}_{\text{g,z}}\text{×test}\text{ }\text{(}\text{w}\text{)} \right)$	(3)

where, $\text{F}_{\text{g}\text{,}\text{r}}$ and $\text{F}_{\text{g}\text{,}\text{z}}$ are the radial and axial components of gravity force, respectively. $\text{test}\text{ (}\text{u}\text{)}$ and $\text{test}\text{ (}\text{w}\text{)}$ are test functions related to the radial and axial velocity components, respectively.

To calculate the relative velocity on a surface, the following *Equation* (4) is used:

$\text{V}_{\text{relative}}\text{ =}\text{ }\text{v}_{\text{r}}\text{×}\text{n}_{\text{r}}\text{+}\text{v}_{\text{∅}}\text{×}\text{n}_{\text{∅}}\text{+}\text{v}_{\text{z}}\text{×}\text{n}_{\text{z}}$	(4)

where, $\text{v}_{\text{r}}$,$\text{v}_{\text{∅}}$ and $\text{v}_{\text{z}}$ are the components of relative velocity in radial, azimuth and axial direction respectively; $\text{n}_{\text{r}}$, $\text{n}_{\text{∅}}$, and $\text{n}_{\text{z}}$ are the components of the grid normal vector in these directions, respectively.

To describe the effect of volume forces on a fluid, the following *Equation* (5) is used:

$$\text{W}_{\text{volume}}\text{=2π}\text{r}\text{ }\left( \text{F}_{\text{v,r}}\text{× test}\text{ }\text{(}\text{u}_{\text{r}}\text{)+}\text{F}_{\text{v}\text{,∅}}\text{×test}\text{ }\text{(}\text{u}_{\text{∅}}\text{)+}\text{F}_{\text{v,z}}\text{×test}\text{ }\text{(}\text{u}_{\text{z}}\text{) } \right)$$

(5)

where, $\text{F}_{\text{v}\text{,}\text{r}}$,$\text{ }\text{F}_{\text{v}\text{,}\text{∅}}$ and $\text{F}_{\text{v}\text{,}\text{z}}$ are the components of volume force in radial, azimuth and axial direction respectively. $\text{test}\text{ (}\text{u}_{\text{r}}\text{), }\text{test}\text{ (}\text{u}_{\text{∅}}\text{) }$and $\text{test}\text{ (}\text{u}_{\text{z}}\text{)}$ are test functions related to the velocity components in these directions, respectively.

To calculate the magnitude of the electrostatic force, the following *Equation* (6) is used:

$\text{F}_{\text{es }}\text{=}\sqrt{\left( {\text{F}_{\text{es}_{\text{z}}}}^{\text{2}}\text{+}{\text{F}_{\text{es}_{\text{r}}}}^{\text{2}} \right)}$	(6)

where $\text{F}_{\text{es}_{\text{z}}}$ and $\text{F}_{\text{es}_{\text{r}}}$ are the axial and radial components of the electrostatic force, respectively.

For the quantitative analysis of the simulation results, the two-dimensional visualization results of the simulation were first exported in the form of an Excel data table [5]. Each position coordinate of the two-dimensional visualization diagram has corresponding x and y values, and corresponding data values. The data of the two-dimensional visualization diagram were taken at simulation times of 0 s, 0.0005 s, 0.0015 s, 0.0025 s, 0.0035 s, 0.0045 s, 0.0055 s, 0.0065 s for velocity, electrostatic force, and the volume ratio of the Taylor cone. The average values of the simulation data points were calculated and used as the Y-axis, with the simulation time as the X-axis, to analyze the change in simulation data over time.

**Optical microscopy images**

An optical microscopy (LEICA DMi1), equipped with FLEXACAM C1 camera, was used to observe the morphology of different jetting fibers. The fibers were collected on a microscope slide and then examined using an inverted microscope at a magnification of 400 ×. The images were subsequently enlarged to four times their original size.

**Scanning electron microscopy (SEM)**

SEM images were collected on a field-emission scanning electron microscope (Hitachi SU-70) with an operating voltage of 5 kV and an operating electricity of 100 pA. Before imaging, the wrap were first coated with Pt by ion sputter coater (ISC 150, China). Fiber diameters on SEM images were measured using ImageJ software (National Institutes of Health). For each sample, the fiber diameters (*n* ≥ 100) were measured.

**Fourier-transform infrared spectroscopy (FT-IR) test**

The interactions between the components of samples were evaluated by FT-IR (Thermo-iS50, USA). Spectra were recorded with a resolution of 4 cm^–1^ with eight-times cyclic scans in the range of 4000 ~ 650 cm^–1^.

**Differential scanning calorimetry (DSC)**

The thermal properties were carried out by a differential scanning calorimeter (Mettler Toledo, DSC3+), as shown in Figure 5. Approximately 2 mg sample was encapsulated in aluminum hermetic pans. A cyclic heating and cooling scan was performed from 20 °C to 200 °C at a heating rate 10 °C·min^-1^ and a cooling rate of 10 °C·min^-1^. The DSC heating curve showed the narrow peak ranging from 45 °C to 65 °C, corresponding to melting of the PCL/CS film crystalline domains. The enthalpy required for melting the crystalline domains per unit mass of the PCZ wrap H_crystalline_ can be estimated by integrating the endothermic transition ranging from 45 °C to 65 °C.

Therefore, the mass of the crystalline domains (*m*_crystalline_) can be calculated as following *Equation* (7):

$\text{m}_{\text{crystalline}}\text{ = }\text{m}_{\text{0}}\text{×}\left( \frac{\text{H}_{\text{crystalline}}}{\text{H}_{\text{crystalline}}^{\text{0}}} \right)$	(7)

where $\text{H}_{\text{crystalline}}^{\text{0}}$ = 138.6 J·g^-1^ is the enthalpy of melting of PC (without zeolite), *m*_0_ is the total mass of the sample.

**SAXS and WAXS test**

To probe the crystal dimension, distance between crystalline domains, interlamellar spacing, as well as the change in the crystalline domains during the PCZ fabrication, light scattering measurements were conducted. SAXS measurements were carried out using the BL16B1 beamline (Shanghai Synchrotron Radiation Facility, SSRF, Shanghai, China), with an X-ray radiation wavelength of 0.1239 nm.

Two-dimensional (2D) SAXS measurements were collected *via* an X-ray detector of Pilatus 2M, with a resolution of 2048 × 2048 pixels. WAXS measurements were carried out using the HomeLab system (Rigaku, Japan), with a Cu-Kα radiation wavelength of 1.54060 Å. Two-dimensional (2D) SAXS were collected via an X-ray detector of HyPix-6000, with a resolution of 1000 × 1000 pixels. The sample holder was mounted onto an optical table, and the sample-to-detector distance was set at 2003 mm for SAXS and 687.9 mm for WAXS. They SAXS and WAXS image acquisition time of each data frame was set at 15 s and 10 s, respectively.

The efficient scattering range of *q* was 0.002 ~ 0.820 nm^−1^ for the SAXS measurements, and the efficient diffraction angle range of 2*θ* was 0 ~ 50° for the WAXS measurements. The measured scattering intensity *I* of the wrap at the swollen state was corrected by subtracting the water and air background. Quantification of SAXS and WAXS patterns was performed with scattering vector (*q*) and azimuthal angle (2*θ*) as coordinates. As previously reported SAXS was used to characterize the inter-crystal spacing, and WAXS was utilised to characterize the crystal dimension, inter-lamellar spacing and inter-molecular spacing [6, 7]. The inter-crystal spacing (*L*_4_) of PCZ wrap was quantified by the one-dimensional scattering curve of corrected scattering intensity (*Iq*^2^) versus scattering vector (*q*) obtained by 2D SAXS patterns, as following *Equation* (8) and (9):

$\text{L}_{\text{4}}\text{=}\frac{\text{2π}}{\text{q}}$	(8)

$\text{q}\text{=}\frac{\text{4πsin}\text{θ}}{\text{λ}}$	(9)

where *λ* is the diffraction wavelength. The average crystal dimension (*D*) of PCZ material was quantified by the one-dimensional scattering curve of intensity versus diffraction angle (2*θ*) obtained by the reflection in WAXS patterns, calculated using the following Scherrer’s *Equation* (10):

$\text{D}\text{=}\frac{\text{kλ}}{\text{β}\text{cos}\text{θ}}$	(10)

where *β* is the half width of the maximum diffraction peak, and *k* is the dimensionless shape factor. The inter-molecular spacing (*L*_2_) and the inter-lamellar spacing (*L*_3_) were determined from the one-dimensional scattering curve of intensity versus scattering vector (*q*) obtained by 2D WAXS patterns, using *Equations* (8) and (9).

**Thermogravimetric analysis (TGA)**

TGA was performed on a TGAⅡ thermogravimetric analyzer (Mettler Toledo, Switerland), as previously reported [8]. The samples were heated to 105 °C and pre-dried for 20 min, then were further heated to 500 °C (10 °C·min^–1^) in a nitrogen atmosphere (25 mL·min^–1^) to investigate the thermal stability of the samples. For that the organic components were completely decomposed. Mineralization content in the wrap was calculated as following *Equation* (11):

$\text{W}_{\text{miner,s}}\text{ = }\left( \text{W}_{\text{inorg,s}}\text{−}\text{W}_{\text{inorg,0}} \right)/{{\left( \text{W}_{\text{inorg,s}}\text{+}\text{W}_{\text{inorg,s}} \right)\text{×100\%}}_{\text{ }}}$	(11)

where $\text{W}_{\text{miner}}$, and $\text{W}_{\text{inorg}}$ were the percentages of minerals and inorganic. $\text{W}_{\text{miner}\text{,}\text{s}}$ and $\text{W}_{\text{inorg}\text{,}\text{s}}$ represented the minerals and inorganic contents for PCZ wraps. $\text{W}_{\text{inorg}\text{,0}}$ represented the inorganic content of PC (without zeolite). For each data point, three samples were measured in parallel, and the final data were presented as mean ± S.D.

**Mechanical tensile test**

Tensile properties of PCZ wrap were measured using the Tinius Olsen H5KT tester. The sample size was 50 mm (length) × 10 mm (width). The samples were stretched at a constant test speed of 10 mm min^-1^ until rupture [9].

**Quasi-static puncture test**

ASTM F3007 was employed for the quasi-static puncture test. The dimension of the wrap samples were 40 mm (length) × 40 mm (width) × 0.33 mm (thickness). For each types of wraps, 3 ~ 5 samples were tested, following a previously reported protocol [10]. The samples were then fixed between two steel hoops (Figure 3d). The puncture tests were conducted using a universal testing machine (MTS systems, CMT6202), as shown in Supplementary Video. 2. A loading nose with a spherical tip (stainless steel, radius = 4 mm) was slowly penetrated through the center of the samples at a quasi-static velocity of 10 μm·s^−1^ until failure. The puncture force (*F*) and puncture displacement (*D*) were measured during the puncture tests (as shown in Figure 3c), and the energy to puncture (*E*) of each panel was determined by computing the area under the force-displacement curves, as following *Equation* (12):

$\text{E}\text{ }\left( \text{D}_{\text{max}}\text{, }\text{F} \right)\text{=}\int_{\text{0}}^{\text{D}_{\text{max}}} \text{sd}\text{D}$	(12)

where *D*_max_ is the maximal displacement until failure.

**Water contact angle (WCA) observation**

The surface wettability of the electrospun fibrous mats was characterized by measuring the water contact angle (WCA) using a contact angle goniometer (SDC-100, SINDIN, China) under ambient laboratory conditions (25 ± 2 °C and 50 ± 5% RH). A droplet of distilled water (3.5 μL) was carefully dispensed onto the mat surface using a calibrated micro-syringe. To minimize dynamic effects and ensure consistent droplet morphology, the contact angle was recorded within 5 s of droplet placement. Contact angles were measured on both the left and right sides of the droplet using image analysis software integrated with the goniometer system [11]. For each sample, measurements were taken at three randomly selected locations across the mat surface to account for surface heterogeneity. The average value and standard deviation were calculated and reported to ensure reproducibility and statistical reliability.

**Ethylene adsorption isotherms**

The ethylene adsorption isotherms were performed at 77 K in an Autosorb 3B, Quantachrome equipment to determine the apparent surface area and the micropore volume using the brunauer-emmet-teller (BET) and Dubinin–Radushkevich models, respectively [12].

The BET test theory is based on the multimolecular layer adsorption model proposed by the trio of Hironol, Emmett, and Taylor, and derives from following *Equation* (13) relating the relationship between monolayer adsorption, *V*_m_, and multilayer adsorption, *V*.

$\frac{\text{P}}{\text{V}\left( \text{P}_{\text{0}}\text{−}\text{P} \right)}\text{=}\frac{\text{1}}{\text{V}_{\text{m}}\text{×C}}\text{+}\frac{\text{C}\text{−1}}{\text{V}_{\text{m}}\text{×C}}\text{×}\frac{\text{P}}{\text{P}_{\text{0}}}$	(13)

where *P* is nitrogen partial pressure, *P*_0_ is saturated vapor pressure of nitrogen at adsorption temperature, *V* is actual adsorbed amount of nitrogen on the sample surface, *V*_m_ is saturated adsorption amount of nitrogen in a single layer, and *C* is constant related to the adsorption capacity of the sample.

**Molecular dynamics simulations of adsorbed ethylene**

All the (DFT) calculations were conducted based on the Vienna Ab-inito Simulation Package (VASP). The exchange-correlation effects were described by the Perdew-Burke-Ernzerhof (PBE) functional within the generalized gradient approximation (GGA) method [13, 14] The core-valence interactions were accounted by the projected augmented wave (PAW) method. The energy cutoff for plane wave expansions was set to 400 eV. The structural optimization was completed for energy and force convergence set at 1.0 × 10^-5^ eV and 0.02 eV·Å^-1^, respectively. The Brillouin zone was sampled with the 2 (length) × 2 (width) × 3 (thickness) K-point. Grimme’s DFT-D3 methodology was used to describe the dispersion interactions. The adsorption energies (*E_ads_*) of C_2_H_4_ molecule are calculated as following *Equation* (14):

$\text{E}_{\text{ads }}\text{=}{\text{E}\text{∗}}_{\text{C}_{\text{2}}\text{H}_{\text{4}}}\text{−}\text{E}_{\text{C}_{\text{2}}\text{H}_{\text{4}}}\text{−}\text{E}_{\text{Sub}}$	(14)

where $\text{E}_{\text{C}_{\text{2}}\text{H}_{\text{4}}}$ and ${\text{E}\text{∗}}_{\text{C}_{\text{2}}\text{H}_{\text{4}}}$ represent the energies before and after the adsorption of C_2_H_4_ molecule on the substrate, respectively. $\text{E}_{\text{Sub}}$ is the energy of clean surface. The electrostatic potential of C_2_H_4_ molecule was calculated using the Gaussian 09 suite of programs. Structural optimization and electronic properties were performed using the B3LYP functional with the def2-TZVP basis set.

**Ethylene Adsorption Test**

First, cut the PCZ wrap into rectangular strips measuring 1 cm × 5 cm, ensuring the surface was clean and undamaged. Prior to testing, samples were equilibrated in a dry environment for 24 hours. Then they were immersed in an ethylene solution (40% aqueous) for varying durations (0, 5, 30, 60, 120 minutes). A high-precision digital multimeter (DT9205A, Germany) was used to measure the real-time resistance value (R) of the PCZ membrane. Finally, a curve plotting *R* versus time was generated.

**Water vapor permeability (WVP)** **measurement**

The WVP measurement of PCL/CS/Zeolite wrap was carried out using a standard test method (GB/T Standard 12704.1, 2009). Circular films previously balanced at 50% RH for 24 h were used to seal the glass cups with a 5.5 cm inside diameter containing 50 mL of deionized water and placed in a dryer containing desiccative silica gel (0% RH). Circular samples were allowed to balance for 2 h before the test bottles were weighed for the first time. The test bottles were weighed with an analytical balance (± 0.0001 g, BSA224S-CW, Sartorius, Germany) every 2 h for 12 h. The slope of weight loss with time obtained by linear regression (r^2^ ≥ 0.99) was used to calculate the water vapor transmission rate (*WVTR,* g·h^-1^·m^-2^). Figure 3i showed a comparison with other reported literatures [15-19] The ultimate *WVP* of the film was calculated as following *Equation* (15):

$\text{WVT}\text{=}\frac{\text{Δ}\text{m}\text{−Δ}\text{m}\text{’}}{\text{A}\text{·}\text{t}}$	(15)

where *Δm* is the difference between two weighings of the same test assemblage. *Δm*’ is the difference between two weighings of the same test assemblage for a blank sample. *A* is the effective test area (0.00283 m^2^ for devices). *t* is the test time (*h*).

**Air permeability (AP)**

The air permeability (AP) of the films was evaluated using a fully automated air permeability tester (YG46E-III, Ningbo Textile Instrument Factory, China) in accordance with standard testing protocols for porous films. A film specimen was fixed in the testing chamber with a defined effective area of 20 cm^2^, and a constant differential pressure of 100 Pa was applied across the film. The resulting air flow rate was recorded and expressed in units of cm^3^·(cm^2^·s)^-1^. Each test was repeated three times on different areas of the film, and the average was reported.

**Biodegradability test**

To assess biodegradability in a natural soil environment [20], rectangular strips (3 cm × 1 cm) of PE and PCZ wraps were buried at a depth of approximately 2 – 3 cm in natural, moist garden soil housed in plastic containers (diameter = 75 mm). The soil moisture was maintained by daily watering with distilled water to simulate environmental humidity. The containers were kept at ambient room temperature (25 ± 2 °C). At monthly intervals, the films were gently removed, cleaned with deionized water, and air-dried before visual inspection and quantification of residual surface area using ImageJ software. The percentage of degradation was calculated based on the initial and remaining surface area.

**Antimicrobial activity evaluation**

The antimicrobial activity of PCZ wraps was evaluated using the Kirby–Bauer disk diffusion method [21], against *Escherichia coli* (ATCC 25922) and *Staphylococcus aureus* (ATCC 25923). Bacterial suspensions were prepared by culturing in brain heart infusion broth at 37 °C for 24 h, and adjusted to a turbidity of 0.5 McFarland standard (~ 1–2 × 10^8^ CFU·mL^-1^) using sterile saline (0.9% NaCl). Petri dishes containing Mueller–Hinton agar were inoculated by lawn spreading of bacterial suspensions. Circular test samples (6 mm in diameter) were cut from PCZ films and aseptically placed on the agar surface. Plates were incubated at 37 °C for 24 h, and the diameter of the inhibition zone around each sample was measured using a digital caliper. All tests were performed in triplicate.

**Plant virus resistance assay**

To assess antiviral performance, the PCZ wrap was applied directly to the left half of tobacco leaves, which were then mechanically inoculated with 500 ng·mL^-1^ *tobacco mosaic virus* (*TMV*) in phosphate-buffered saline (PBS). The virus solution was gently rubbed into the leaf surface using a gloved finger, ensuring uniform inoculation. The right half of the same leaf served as the untreated internal control. After inoculation, the leaves were gently rinsed with PBS to remove unbound virus, and plants were incubated under controlled growth conditions (25 ± 2 °C, 16 h light/8 h dark) for 24 h in a growth chamber. Subsequently, leaves were detached, and localized lesions were counted to determine TMV infectivity. The average lesion count from each condition was compared with controls to assess virus inhibition [22].

**Transmission electron microscope (TEM) imaging observation**

The PCZ wrap extract (positive control) was mixed with TMV. The blank control (CK) consisted of a solution containing TMV particles without any compound. The sample solution was adsorbed on a 200 mesh copper plate, mixed for 30 min and counterstained with 1% phosphotungstic acid. After drying, the morphology of the TMV particles was observed and micrographs were taken and all images recorded using transmission electron microscopy (Tecnai G2 F20 S-TWIN, USA). The antiviral rate was calculated using *Equation* (16):

$\text{Antiviral}\text{ \% =}\left( \text{1}\text{−}\frac{\text{Number of spots in PCZ group}}{\text{Number of spots in the control group}} \right)\text{×100\%}$	(16)

For each experiment, the antiviral rate was determined based on the TMV spot counts in the membrane and control groups. The mean antiviral rate across all experiments was then calculated using *Equation* (16) to provide an overall assessment of antiviral efficacy. Additionally, the standard deviation of the antiviral rates was calculated to indicate the variability and reproducibility of the experimental data.

**Plant biosafety evaluation**

To assess the phytotoxicity of the PCZ wrap, Pisum sativum (field pea) seeds were soaked in deionized water and germinated in an experimental greenhouse under controlled ambient conditions. After germination, uniform bean sprout seedlings were transferred into a culture tank containing PCZ wrap extract at a concentration of 1 mg·mL^-1^, while seedlings grown in tap water served as the control group. The culture tanks were maintained at 25 ± 2 °C under a 16/8 h light/dark photoperiod. Observations were recorded on Day 0, 2, 9, and 12, and plant growth parameters, including average germination rate and plant height, were measured. Each treatment was performed in triplicate.

**Fish biosafety evaluation**

## Adult zebrafish (wild-type, AB strain) was obtained from the China Zebrafish Resource Center (Wuhan, China). All zebrafish seemed healthy based on their appearance and physical characteristics, with an average body length of 2.7 ± 0.2 cm. All animal procedures strictly adhered to international ethical guidelines and were reviewed and approved by the Ethics Committee of Shenzhen Guangming District People's Hospital (Approval No.: LL-KT-2024110). The water used for acclimation was pre-aerated for 12 h to ensure sufficient oxygen, and was maintained at a temperature of 25 ± 1 °C, pH of 7.0 ~ 7.4. The zebrafish culture medium was slightly modified according to the U.S. Environmental Protection Agency (EPA) Toxicity Guidelines for aquatic organisms [22]. The zebrafish domesticated for 15 days were placed in blank culture medium (75 mm in diameter) and culture medium containing 10 mg of PCZ wrap. In this work, PCZ wrap with a same diameter of culture medium (75 mm) was approximately 15 mg. Each treatment was replicated three times, with each replicate containing seven zebrafish. The zebrafish were cultured with a day/night cycle of 16:8 h without feeding during exposure. The zebrafish were monitored using videos, and viability was calculated at 3, 5 and 7 days [23].

**Animal breathing biosafety evaluation**

BALB/c mice used in this study were purchased from Beijing VTLH Laboratory Animal Technology Co., Ltd. All mice were male, 8 weeks old, and weighed (20±2 g). Animals were housed in an SPF-level barrier environment at the Agricultural Genomics Institute at Shenzhen Laboratory Animal Center and acclimated for at least 7 days. Housing conditions were maintained at 25±2°C, 55±10% relative humidity, with a 12-hour light/12-hour dark cycle. Animals had free access to standard irradiated sterilized feed and sterile drinking water. All animal procedures strictly adhered to international ethical guidelines and were reviewed and approved by the Ethics Committee of Shenzhen Guangming District People's Hospital (Approval No.: LL-KT-2024110). This study also followed the Declaration of Helsinki. Mice were randomly assigned to either the PCZ group or the blank control group, with 3 independent replicates per group, each replicate comprising 2 mice (total *n* = 6 per group). Mice were randomly assigned to either the PCZ film combustion exposure group or the blank control group. Exposed mice were individually placed in a sealed exposure chamber where 1g of PCZ packaging film was ignited and allowed to burn completely. Mice were exposed to the combustion-generated gases for 2 minutes before immediate transfer to a clean, fresh-air environment for continued observation for 24 hours. Blank control mice were placed in the sealed chamber under identical conditions without combustion. At the end of the observation period, mice were euthanized via carbon dioxide inhalation followed by cervical dislocation to minimize suffering. Immediate dissection was performed to harvest cardiac, hepatic, splenic, pulmonary, and renal tissues from each mouse. Tissue blocks were fixed in 4% paraformaldehyde, paraffin-embedded, sectioned, and stained with hematoxylin and eosin. Histopathological alterations in each organ were examined under a light microscope.

**Cell proliferation**

To evaluate the inherent cytocompatibility of the PCZ dressing material, its effect on the proliferation of mouse fibroblast (L929) cells was assessed using the Cell Counting Kit-8 (CCK-8) assay. The L929 cells line was obtained from our laboratory culture. Cells were routinely maintained in RPMI-1640 medium supplemented with 10% (v/v) fetal bovine serum (FBS) and 1% (v/v) penicillin/streptomycin at 37 °C in a humidified atmosphere containing 5% CO₂. The PCZ wrap was cut into samples measuring 10 × 10 × 0.3 mm (length × width × thickness), sterilized under UV light for 30 minutes, and pre-wetted with complete culture medium for at least 1 hour prior to cell seeding. L929 cells were seeded at a density of 2 × 10⁵ cells per sample onto the pre-treated PCZ wrap placed in individual wells of a 24-well plate. Cells seeded directly onto the tissue culture plastic of a 24-well plate (without any material) served as the positive control group. After co-culture periods of 1, 3, and 5 days, the PCZ wrap with adherent cells were carefully retrieved using sterile forceps, gently rinsed twice with phosphate-buffered saline (PBS) to remove non-adherent cells, and transferred to a new 24-well plate. Then, 300 µL of fresh culture medium containing 10% (v/v) CCK-8 reagent was added to each well containing a sample. The plate was incubated at 37 °C for 30 minutes to allow for formazan dye formation. Following incubation, 100 µL of the reaction solution from each well was carefully aspirated and transferred to the corresponding wells of a 96-well plate. The absorbance of each well was immediately measured at a wavelength of 450 nm using a microplate reader (Shimadzu Corporation, Kyoto, Japan).

**Fresh-keeping effect evaluation**

The cherries and tomatoes used in this study were purchased from local supermarkets in Shenzhen, China (see Materials and chemicals section for details). To ensure experimental consistency, all fruits underwent a rigorous selection process. Cherries and tomatoes were first chosen based on their good appearance, intact condition, and absence of visible mold or rot. The specific selection criteria for cherries were as follows: fruits must retain intact stems, exhibit no bruises, cuts, or blemishes on the skin, demonstrate uniform size and weight, and display optimal bright red coloration, indicating commercial ripeness. Similarly, tomatoes with missing stems or visible wounds were excluded. Only those with comparable size, weight, and uniform color were selected. All selected fruits were rinsed with deionized water for 30 minutes to remove surface impurities and pesticide residues. After gently patting them dry with clean paper towels, the fruits were air-dried at ambient temperature (25 ± 2°C). After processing, the fruits were divided into experimental groups. The cherry tomato groups were packaged and stored at 25 ± 2°C, with analyses conducted at designated time points (0, 3, and 7 days of storage).

**Firmness test**

Fruit firmness was evaluated using a Precision Universal Testing Machine (AGS-10kNXD). For each fruit (cherry and tomato), a compression test was performed in three repeated cycles using an 8 cm-diameter probe at a constant speed of 1.5 mm·s^-1^. The peak force during the first cycle, corresponding to the burst of the fruit’s pericarp, was recorded as the burst point. The average force across three replicates was used for comparison.

**Weight loss rate measurement**

During the storage period, the weight of each fruit sample was measured at a specific time (*W_t_*). Figure 5i showed a comparison with other reported literatures [24-34]. The weight loss rate (*W*) of the fruit, presented as a percentage, was then calculated using *Equation* (17):

$\text{W}\text{ \% =}\left[ \left( \text{W}_{\text{0}}\text{−}\text{W}_{\text{t}} \right)\text{÷}\text{W}_{\text{0}} \right]\text{×100\%}$	(17)

where *W_0_* represents the initial weight of the sample.

**Color analysis**

The chroma values are measured by a calorimeter (TS7000, 3nh Co., Ltd., Shenzhen, China). The color was measured at three equidistant points on each fruit, and *L** (light/dark), *a** (red/green) and *b** (yellow/blue) were recorded at 0, 3 and 7 days. The color change (Δ*E*) was calculated, as following *Equation* (18):

$\text{Δ}\text{E}\text{=}\sqrt{{\text{Δ}\text{L}}^{\text{2}}\text{+}{\text{Δ}\text{a}}^{\text{2}}\text{+}{\text{Δ}\text{b}}^{\text{2}}}$	(18)

where Δ*L* is the differential value of *L* of the wraps, Δ*a* is the differential value of a of the wraps, and Δ*b* is the differential value of *b* of the wraps. The differentials were calculated between the control wrap and wrap sample.

**POD enzyme activity and reducing sugar content (RSC)**

Each fruit sample (cherry and tomato) (5 g) was weighed and mixed with 5 mL POD extraction buffer (1 mmol·L^-1^ ethylene diamine tetraacetic qcid, 1 mmol·L^-1^ phenylmethylsulfonyl fluoride, 4 mmol·L^-1^ dithiothreitol, 3% crosslinked polyvinylpyrrolidone and 10 μmol·L^-1^ pyridoxal phosphate) and 5 mL RSC extraction buffer (10% glycerine, 5% crosslinked polyvinylpyrrolidone, 5.0 mmol·L^-1^ dithiothreitol, 30 mmol·L^-1^ L-Ascorbic acid sodium salt, 0.1 mmol·L^-1^ FeSO_4_), respectively. The homogenate was then ground in an ice bath and centrifuged at 12,000 × g at 4 °C for 30 min. The supernatant was collected and used as an enzyme extract. The POD enzyme activity and RSC were determined by Solarbio ELISA Kit (Beijing, Solarbio Life Sciences *Co.*, Ltd., China).

### Metabolomics analysis

### Sample preparation and extraction

Using vacuum freeze-drying technology, place the cherry fruit tissues in a lyophilizer (Scientz-100F), then grinding (30 Hz, 1.5 min) the samples to powder form by using a grinder (MM 400, Retsch). Next, 50 mg of sample powder was weighed using an electronic balance (MS105DΜ), and add 1200 μL of -20 °C pre-cooled 70% methanolic aqueous internal standard extract (less than 50 mg added at the rate of 1200 μL extractant per 50 mg sample). The mixture was vortexed every 30 min for 30 s, a total of 6 times. After centrifugation (rotation speed 12,000 × g, 3 min), the supernatant was aspirated, and the sample was filtered through a microporous membrane (0.22 μm pore size) and stored in the injection vial for UPLC-MS/MS analysis.

### High performance liquid chromatography (HPLC) tests

All samples were acquired by the LC-MS (Liquid Chromatography-Mass Spectrometry) system followed machine orders.The analytical conditions were as follows, UPLC: column, Waters ACQUITY UPLC HSS T3 1.8 µm, 2.1 mm × 100 mm; column temperature, 40 °C; flow rate, 0.40 mL·min^-1^; injection volume, 4 μL; solvent system: water (0.1% formic acid) and acetonitrile (0.1% formic acid). Sample measurements were performed using a gradient program starting at 95% A and 5% B. A linear gradient to 35% A and 65% B was programmed over 5 min. Within 1 min, a linear gradient to 1% A and 99% B was programmed and maintained for 1.5 min. Subsequently, the composition was adjusted to 95% A and 5% B within 0.1 min and held for 2.4 min.

**MS conditions**

The data acquisition was operated using the information-dependent acquisition (IDA) mode using Analyst TF 1.7.1 Software (Sciex, Concord, ON, Canada). The source parameters were set as follows: ion source gas 1 (GAS1), 50 psi; ion source gas 2 (GAS2), 60 psi; curtain gas (CUR), 35 psi; temperature (TEM), 550 °C, or 550 °C; declustering potential (DP), 80 V, or -80 V in positive or negative modes, respectively; and ion spray voltagefloating (ISVF), 5500 V or -4500 V in positive or negative modes, respectively. The TOF MS scan parameters were set as follows: mass range, 50 ~ 1250 Da; accumulation time, 200 ms; and dynamic background subtract, on. The product ion scan parameters were set as follows: mass range, 50 ~ 1250 Da; accumulation time, 40 ms; collision energy, 30 or -30 V in positive or negative modes, respectively; collision energy spread, 15; resolution, UNIT; charge state, 1 to 1; intensity, 100 cps; exclude isotopes within 4 Da; mass tolerance, 50 mDa; maximum number of candidate ions to monitor per cycle, 12.

**Methods of LC-MS metabolomic data processing**

The original data file acquired by LC-MS (Liquid Chromatography-Mass Spectrometry) was converted into mzXML format by ProteoWizard software. Peak extraction, peak alignment and retention time correction were performed respectively by XCMS program. The Support Vector Regression (SVR) method was used to correct the peak area. The peaks with detection rate lower than 50% in each group of samples were discarded. Subsequently, metabolic identification information was obtained by searching the laboratory’s self-built database, integrated public database, AI database and metDNA.

**Principal components analysis (PCA)**

Unsupervised PCA (principal component analysis) was performed using the statistical function prcomp in R (www.r-project.org). The data were scaled to unit variance before unsupervised PCA.

**HCA and PCC analysis**

The HCA (hierarchical cluster analysis) results of samples and metabolites were presented as heatmaps with dendrograms, while pearson correlation coefficients (PCC) between samples were caculated using the cor function in R and presented as only heatmaps. Both HCA and PCC were carried out by R package ComplexHeatmap. In HCA, normalized signal intensities of metabolites (unit variance scaling) were visualized as a color spectrum.

**Metabolite analysis using OPLS-DA and VIP**

For two-group analysis, differential metabolites were determined by Variable Importance Projection (VIP) (VIP > 1) and absolute Log_2_FC (|Log_2_FC| ≥ 1.0) (Supplementary Table S5). VIP values were extracted from OPLS-DA result, which also contain score plots and permutation plots, was generated using R package MetaboAnalystR. The data was log transform (Log_2_FC) and mean centering before OPLS-DA (Supplementary Table S4). To avoid overfitting, a permutation test (200 permutations) was performed.

**KEGG annotation and enrichment analysis**

Identified metabolites were annotated using Kyoto Encyclopedia of Genes and Genomes (KEGG) Compound database (<http://www.kegg.jp/kegg/compound/>), annotated metabolites were then mapped to KEGG Pathway database (<http://www.kegg.jp/kegg/pathway.html>). Significantly enriched pathways are identified with a hypergeometric test’s P-value for a given list of metabolites.

Metabolites are the basis of an organism's phenotype and can help to understand biological processes and their mechanisms more intuitively and effectively. Based on the qualitative and quantitative analysis of metabolites, metabolomics can be used to analyze metabolic pathways or metabolic networks, and to study the metabolic basis of macroscopic phenotypic phenomena in different biological individuals.

Untargeted metabolomics is a commonly used metabolomics research method, the main research idea is to compare the experimental group and the control group, detect the metabolites contained in the samples, and obtain quantitative information, to find the statistically significant difference metabolites between different groups, which can explain the connection between the found metabolites and the biological process or biological state.

In this work, six samples were selected and divided into two groups for metabolism study, and a total of 2,725 metabolites were detected (Supplementary Table S3).

Unit variance scaling (UV), also called Z-score standardization, or auto scaling, is a method of standardizing data based on the mean and standard deviation of the original data. The processed data conforms to the standard normal distribution, *i.e.*, the mean is 0 and the standard deviation is 1. Calculation method: the original data after standardization divided by the variable standard deviation (standard deviation). The formula is as following *Equation* (19):

$\dot{\text{x}}\text{=}\frac{\text{x}\text{ −}\text{μ}}{\text{σ}}$	(19)

where *µ* is the mean and *σ* is the standard deviation.

Centered/Zero-mean Zero-centered (Ctr). Calculated by subtracting the mean value of the variable from the original data. The formula is as following *Equation* (20):

$\dot{\text{x}}\text{=}\text{x}\text{ }\text{−}\text{μ}$	(20)

**Life-cycle assessment (LCA)**

The LCA analysis followed the ISO standard series 14040 and was conducted using OpenLCA 1.10.3. Two functional units were used, one was a constant mass (1 kg) of the PCZ wrap produced, and the other normalized the environmental impact results by tensile strength, allowing comparison of different plastic cling films in terms of both environmental impacts and material properties. The system boundaries included production of various raw materials, transportation and lignocellulosic bioplastic production.

**Model evaluation of PCZ wrap applications**

To assess the global use of PCZ wrap, we developed an environmental impact assessment model based on Life Cycle Assessment (LCA) data [35], population, and 2021 per capita fruit consumption estimates [36-38]. The model assumes a direct proportional relationship between fruit consumption and plastic wrap usage, with 1 kg of fruit consumption equating to 1 kg of plastic wrap. Traditional plastic wrap serves as the reference. We used a demand growth model (*Sigmoid curve*), combined with quantitative analysis of market penetration and volume data, to forecast future market demand. First, we calculated the market capacity for each country by multiplying the population by the annual per capita fruit consumption to arrive at the maximum potential market size. We then used the demand growth model to forecast future market demand, as following *Equation* (21).

$\text{Market}\text{ }\text{demand}\text{ = }\text{Max}\text{ }\text{market}\text{ }\text{demand}\text{×}\left( \frac{\text{1}}{\text{1+}\text{e}^{\text{−}\text{k}\left( \text{t}\text{−}\text{t}_{\text{m}} \right)}} \right)$	(21)

where *e* represents the base of the natural logarithm (approximately 2.71828); *k* represents the growth rate constant; *t* represents the time span from the star year to the predicted year; *t*_m_ indicates the time span during which the demand reaches its maximun value. Using the total consumption data, we calculated the amount of fruit consumed in each country. We then computed the environmental impacts of conventional plastic wrap and PCZ wrap, including Global Warming Potential (GWP), Acidification Potential (AP), and Terrestrial Ecotoxicity Potential (TETP). A comparative analysis was conducted to assess the efficacy of PCZ wrap in reducing carbon emissions, acidification, and ecotoxicity compared to traditional plastic wrap [39]. Finally, we ranked fruit consumption by country, identified the 15 countries with the greatest environmental improvement, and comprehensively evaluated the potential of PCZ wrap in reducing AP and TETP.

R Studio was used to visualize the GWP improvement data, and the rnaturalearth package was used to render the data globally. All relevant code files are available at: <https://github.com/domelxb/PCZ-wrap>.

**Photograph and video recording**

In the absence of specific instructions to the contrary, all video recordings and photographic documentation in this research were executed using a high-resolution camera (SONY, A7R IV, Japan) coupled with a zoom lens.

**Statistical analysis**

All experimental data were expressed as mean ± standard deviation (mean ± SD). Statistical analyses were performed using GraphPad Prism 9.0.0 and Origin 2022 software. Depending on the data structure, either two-tailed Student’s t-tests or one-way analysis of variance (ANOVA) was applied. A significance level of *α* = 0.05 was used, and results were considered statistically significant when *p* < 0.05. Statistical significance was indicated as follows: *p* < 0.05 (*), *p* < 0.01 (**), *p* < 0.001 (***), and *p* < 0.0001 (****).

**S.III. Supplementary results**

Figure S1 | Photography settings for degradation experiments.

Figure S2 | Gel permeation chromatography (GPC) molecular weight distribution of PCL in PCZ ink after treatment with 90% aqueous acetic acid.

Figure S3 | Principles of design and operation for handheld electro-jetting system. (a) The portable system is powered by a rechargeable battery and features four flow rate settings. (b) Solvent evaporation and continuous tapering during fibrous formation when it comes out of the Taylor cone. (c) Electrostatic forces overcome surface tension to create a fibrous coating (fresh-keeping wrap) on the rotating target (Orange). (d) Electro-jetting [system block diagram](https://www.sciencedirect.com/topics/engineering/system-block-diagram). (e) Electronic circuit for electro-jetting device. (f) Operation process of handheld electro-jetting device.

Figure S4 | Jetability and structural analysis. (a) Multiphysics finite element simulation of the jetting process during electro-jetting at different zeolite concentrations and velocities of PCZ inks. Pure solvent with a zeolite concentration of 0 *wt.*% and a velocity of 1.5 mL·h^-1^. PCZ solution with a zeolite concentration of 0.5 *wt.*% and a velocity of 1.5 mL·h^-1^. PCZ solution with a zeolite concentration of 1 *wt.*% and a velocity of 10 mL·h^-1^. The velocity distribution of the jetting process over time illustrates the influence of PCZ concentrations and flow velocities on fluid dynamics. (b) Volume of sprayed solvent at different flow rates. As the Taylor cone formed and stabilized, the electric field strength at the tip of the cone peaks, resulting in a maximum electrostatic force. Over time, the Taylor cone gradually stretched and formed a jet stream, with a subsequent weakening of the electrostatic force. (c) Distribution of fibre diameters. The histogram represents the relative proportion of different fibre diameters for 0.5 *wt.*% PCZ solution electrospun at a flow rate of 5 mL·h^-1^, with a fitted curve indicating the overall diameters’ distribution. (d) Schematic diagram of zeolite release within fibers (e) SEM observation of zeolite distribution in PCZ fibers. (f) Crystallinity tests. DSC thermograms of PCL/CS, PCL/CS/Zzeolite (0.5 *wt.*%), and PCL/CS/Zeolite (1 *wt.*%), indicating differences in crystallization behavior with increasing zeolite concentrations. The insets illustrate the microstructural arrangement of PCL, zeolite, and crystal domains at different stages of the crystallization process. (g) TGA curves of various mineralized wraps. Thermogravimetric analysis (TGA) of PCZ wraps with different zeolite concentrations (0 *wt.*%, 0.5 *wt.*%, 1.0 *wt.*%). The samples were heated at a rate of 10 °C·min^-1^ from 20 °C to 500 °C. Weight loss was observed in three main stages: initial water evaporation, polymer decomposition, and residual inorganic component. The inset schematic illustrates the TGA setup, with heating applied to the sample during the weight loss measurement. (h) Microstructural characteristics of PCZ fibre. Schematic diagram of X-ray scattering characteristic for PCZ fibre analysis. WAXS pattern of the control sample (PCL/CS fibre). SAXS pattern of the control sample (PCL/CS fibre). (i) WAXS analysis indicating crystal size distribution, highlighting uniform dispersion of zeolites in the PCZ fibre compared to the control sample. (j) SAXS intensity profiles of the blank and PCZ fibre samples, showing a shift in peak position and corresponding changes in long-range ordering, suggesting a structural alteration with the addition of zeolites.

Figure S5｜Assessment of tensile mechanical property stability. (a) Statistical analysis of tensile mechanical strength changes after two weeks. (b) Statistical analysis of tensile mechanical toughness changes after two weeks. Data are presented as mean ± SD, *n* = 3.

Figure S6 | Puncture resistance, intermolecular interaction, biodegradablity and biocompatibility evaluation of PCZ wrap. (a) 3D topography of the PCZ wrap surface after puncture testing, showing the highest and lowest points and the direction of puncture force. (b) Top view of the PCZ wrap surface, highlighting the height distribution. (c) Height profile along the punctured region, indicating the depth of depression between the highest and lowest points. The maximum puncture distance is 1066.6 µm. (d) Tensile strength and toughness of the PCZ wrap measured after storage at Env-1 (25 °C, 50% RH) and Env-2 (40 °C, 75% RH) for 0, 3, and 7 days. (e) Water contact angles of different film compositions, showing improved hydrophobicity with zeolite incorporation. (f) Molecular dynamics simulation. Top view of the charge density difference of ethylene molecules adsorbed in the zeolite space structure. Yellow and blue lobes indicate charge accumulation and charge separation regions. In the charge separation regions, gray, orange, and blue spheres represent aluminum, silicon, and oxygen atoms, respectively. (g) RDG *vs* sign (*λ*_2_) *ρ* plot from IGM/IGMH analysis for the ethylene-zeolite system. The color scale indicates interaction nature: blue (negative) attractive, green (near zero) *van der Waals*, and red (positive) steric repulsion. Data in (d) are means ± SD, *n* = 3.

Figure S7 | Electrical resistance response of PCZ wrap upon exposure to ethylene (C₂H₄). The inset schematic illustrates the experimental setup for resistance measurement during ethylene exposure. Data are presented as mean ± SD, *n* = 3.

Figure S8 | (a) Biodegradability evaluation of PE wrap and PCZ wrap in natural soil. Biodegradability test of PE wrap and PCZ wrap in natural soil over a 6-month period. The area of each wrap was measured monthly, showing a significant reduction in the PCZ wrap, which fully degraded by month 3, while the PE wrap remained mostly intact. The degradation process for the PCZ wrap started from month 1, with the dashed line indicating the trend towards complete degradation. (b) Effect of PCZ wrap incineration gas on respiration of mice. Histological observation (H&E staining) of mice (Blank) heart, liver, spleen, lung and kidney. Data in (a) are means ± SD, *n* = 3.

Figure S9 | Histopathological section analysis of organs such as the liver (a) and kidneys (b) in zebrafish.

Figure S10 | Evaluation of fruits quality. (a) Evaluation of tomatoes quality on different storage conditions. Comparative discernible appearance changes in tomatoes with and without PCZ wrap over a 7-days period. (b) Decay rate of tomatoes model. Comparison of the decay rate of tomatoes over different storage time (days) between the blank (without PCZ wrap) and PCZ wrap groups. (c) Mangoes and (d) bananas were individually wrapped using tinfoil, polyethylene (PE), or PCZ wrap and stored under ambient conditions. Compared to tinfoil and PE, which showed significant browning, spotting, and softening by Day 7, PCZ wrap effectively delayed visible spoilage, minimized surface deterioration, and preserved the original color and texture. (e) Representative photographs of banana, mango, tomato, and cherry during storage (Day 0, Day 3, and Day 7). (f) Weight loss rates of different fruits at Day 3 and Day 7. (g) Firmness of the tomato skin measured at different storage time, and puncture energy of the tomatoes on Day 0, 3, and 7. (h) Weight loss rate of tomatoes after 3 and 7 days of storage time, showing significant difference between the blank and PCZ wrap groups (****p* < 0.001). Data in (f), (g) and (h) are means ± SD, *n* ≥ 3.

Figure S11 | Preservation performance of PCZ wrap on fruits with naturally waxy epidermal layers. (a) Representative photographs of pears and apples wrapped with PCZ wrap during storage at Day 0, Day 1, Day 3, and Day 7. (b) Quantitative comparison of weight loss rates of pears and apples wrapped with PCZ wrap at Day 3 and Day 7. Data are presented as mean ± SD, *n* = 3.

Figure S12 | Evaluation of tomatoes quality. (a-d) Evaluation of color stability and biochemical integrity of tomatoes during storage. Changes in color parameters (*L**, *a**, *b**, and total color difference Δ*E*) of tomatoes stored with and without PCZ wrap at Days 0, 3, and 7. The results indicate the effectiveness of PCZ wrap in maintaining color stability during storage. (e) Peroxidase (POD) activity in tomatoes, illustrating enzymatic degradation over time, with lower activity observed in PCZ wrap samples. (f) Reducing sugar content (RSC) of tomatoes stored at 25 °C for 0, 3, and 7 days, showing improved preservation with PCZ wrap. Data are presented as mean ± SD, *n* ≥ 3.

Figure S13 | Changes in color parameters (Δ*L**, Δ*a**, Δ*b**) for banana (a), mango (b), cherry (c), and tomato (d) during storage. (e, f) Surface observation of SME after 7 days of banana preservation using PCZ wrap. Data are presented as mean ± SD, *n* = 5.

Figure S14 | Full-chain evaluation of PCZ wrap. (a) A large-scale commercial fruit preservation using PCZ wrap prepared by a portable electrostatic jetting system ensures quality preservation throughout the entire fruit supply chain, from the farmer's pick to the customer's enjoyment. (i) Fresh fruit is easily picked into orchard by farmers. (ii-iii) Farmers readily employ the portable electro-jetting system to fabricate the PCZ wrap, ensuring the fruit is precisely covered. (iv) The PCZ wraps can be prepared quickly and in large quantities. (v-vi) The fresh fruits are then packaged and transported. (b) (vii) Bulk transportation to next destination. (viii-ix) The fruits with PCZ wraps are intactly delivered to the customers. **(**c) (x) The PCZ wrap features easily tear-off and bacteriostatic properties. (xi) It leaves no residue on the surface of food and is easy to clean up. (xii) Customers enjoy the fresh and healthy food at home.

Figure S15 | Quantitative evaluation of spraying efficiency and coating uniformity of the handheld electrostatic deposition (HED) device on representative fruits. (a) Relationship between spraying time and coated surface area for tomato, cherry, mango, and banana under a constant flow rate (*V* = 5 mL·h⁻¹), as operated by an adult and a 10-year-old child. (b) Thickness of the deposited PCZ coating on different fruits measured after spraying by adult and child operators. No statistically significant differences (ns) were observed between groups. Data are presented as mean ± SD, *n* = 3.

Figure S16 | Non-targeted metabolomics analysis for post-treated quality evaluation. **(**a) Correlation analysis of quality control (QC) samples. Pearson correlation analysis was performed on the QC samples. The closer the correlation coefficient (|r|) is to 1, the better the stability of the testing process and the higher the quality of the data. (b) OPLS-DA S-plot showing significant metabolic differences between cherries treated with PCZ wrap and untreated blank group. Metabolites positioned near the top right and bottom left indicate the greatest differences. Yellow points represent metabolites with VIP values greater than 1, emphasizing their significant contribution to group separation, while green points indicate metabolites with VIP values ≤ 1. (c) Dynamic distribution of metabolite fold changes (log_2_FC) in the comparison group, illustrating overall metabolic differences. Metabolites were ranked from lowest to highest based on FC values. The top 10 upregulated and downregulated metabolites are labeled to highlight significant changes, with metabolites showing the highest positive changes ('Top up') and those with the greatest negative changes ('Top down'). (d) VIP value map of differential metabolites. Top 20 differential metabolites based on VIP scores from the OPLS-DA model. Upregulated metabolites are shown in red, downregulated in green, indicating their importance in distinguishing between groups. (e) Differential metabolite correlation analysis. The horizontal and vertical axes represent the names of the differential metabolites. Different colors represent the pearson correlation coefficients, with the relationship between the coefficients and colors shown in the legend on the right. Red indicates a stronger positive correlation, while green indicates a stronger negative correlation. Darker colors correspond to larger absolute values of the correlation coefficients between the samples. This figure shows the top 50 differential metabolites with the highest VIP values. (f) Differential metabolite Z-value plots. The horizontal axis represents the Z-value, and the vertical axis represents the metabolites. Different colored points represent different groups of samples. This figure shows the top 50 differential metabolites with the highest VIP values. (g) Differential metabolite KEGG classification. The vertical axis represents the names of the KEGG metabolic pathways, and the horizontal axis represents the number of differential metabolites annotated to each pathway, as well as their proportion relative to the total number of annotated metabolites. (h) KEGG pathway enrichment analysis of differential metabolites, showing the top 20 pathways ranked by P-value. Rich Factor represents the ratio of differential metabolites to total metabolites in the pathway. Differential Abundance Score (DA Score) analysis illustrating the overall metabolic changes within each pathway, with up- and down-regulated trends. (i) Heat map with different wraps indicating the comparison of the contribution analysis made to the LCA. The potential impact categories analyzed were: abiotic depletion (ADP), acidification (AP), terrestrial ecotoxicity potential (TETP), eutrophication (EP), global warming (GWP), human toxicity (HTP), and photochemical ozone formation potential (POFP).

**S.IV. Supplementary videos**

**Supplementary Video 1. Simulation of electro-jetting process.**

Multi-physics field coupled modeling was used for the simulation. When the potential difference of the simulation was 15 kV, the whole process of Taylor cone formation was successfully simulated, *i.e.*, the solution overcomes the surface tension and forms an elongated liquid column under the action of electric field force.

**Supplementary Video 2. Quasi-static puncture test of PCZ wrap.**

Quasi-static puncture test of PCZ wrap using a universal testing machine. The video demonstrates the progression of the puncture test, recording force-displacement data and showing deformation until complete penetration. Surface morphology post-puncture highlights the mechanical properties and resilience of PCZ wrap.

**Supplementary Video 3. Fish bio-safety evaluation.**

It was observed that the activity of fishes in petri dishes *co*-cultured with PCZ wrap on day 3, 5 and 7 were not significantly different from the activity of fishes in petri dishes without any treatment. (*n* = 7)

**Supplementary Video 4. Animal breathing bio-safety evaluation.**

Mice were observed to move flexibly in a confined space where gases were released by burning PCZ wrap. No significant changes were observed compared to mice without any treatment.

**Supplementary Video 5. Operation process of handheld electro-jetting device.**

Demonstration video shows operation process of PCZ wrap fabrication for tomatoes *via* a handheld electro-jetting device.

**Supplementary Video 6. PCZ wrap stripping.**

Stripping PCZ wrap from tomatoes showed that it was easy to strip without residue.

**Supplementary Video 7. Cherry firmness test.**

Puncture test was performed on the cherries preserved with PCZ wrap for 7 days to evaluate the firmness of cherry.

**S.V. Appendix**

**Table S1. Detailed information on the fruits used in this study.**

| **Fruit** | **Types of fruit breathing** | **Variety** | **Weight/diameter** | **Origin** |
| --- | --- | --- | --- | --- |
| **tomatoes** | Climacteric | Sweet 100 | 80–150 g | Italy |
| **cherries** | Non-climacteric | Santina | 24.6–26 mm | Chile |
| **bananas** | Climacteric | Williams | 160–180 g | China |
| **mangoes** | Climacteric | Tainong No.1 | 120–140 g | China |

**Table S2. Summarizing the key parameters (such as breathability, degradation time, etc.) of traditional packaging (aluminum foil) and the PCZ wrap.**

| **Degradability** | **Antibacterial Property** | **Breathability** |
| --- | --- | --- |
| Non-degradable | None | None [39] |
| Degradable（≈6 months） | Good Antibacterial Property（ >92% antibacterial efficacy against pathogens like E. *coli* and S. *aureus*） | Good Breathability |

**Table S3. Statistics on the number of metabolites identified.**

| **Ionization mode** | **All** | **T3_positive** | **T3_negative** |
| --- | --- | --- | --- |
| Number of metabolites | 2725 | 1721 | 1004 |

**Table S4. Parameters of OPLS-DA.**

| **Compare group** | **R2X** | **R2Y** | **Q2** |
| --- | --- | --- | --- |
| Control vs PCZ wrap | 0.726 | 1 | 0.961 |

**Table S5. The top 50 differentially metabolized VIP values.** The top 12 significant differences (*****P* < value 0.0001); 13-40 significant differences (****P* < value 0.001); 41-50 significant differences (***P* < value 0.01).

| Index | Compounds | Class I | Class II | score | Control  (Average raw intensity) | PCZ wrap  (Average raw intensity) |
| --- | --- | --- | --- | --- | --- | --- |
| **MW0013113** | 2,3-dinor Thromboxane B1 | FA | Others | 8.69E-01 | 493019.0767 | 13921.53 |
| **MEDN1284** | LPE(18:3/0:0) | GP | LPE | 7.50E-01 | 32078.60333 | 2860.97 |
| **MEDN0224** | Trehalose | Others | Saccharides | 9.59E-01 | 51405.75333 | 194814.0867 |
| **MW0149761** | Gln-Lys-Phe-Arg | Amino acids and derivatives | Amino acids and derivatives | 7.41E-01 | 130176.4633 | 18873.68667 |
| **MEDL02681** | 6-Deoxyfagomine | Alkaloids | Piperidine alkaloids | 9.70E-01 | 92261.25333 | 15153.39333 |
| **MEDP1904** | 1-(9Z,12Z-octadecadienoyl)-glycero-3-phosphoethanolamine | GP | LPE | 7.58E-01 | 175464.54 | 25167.79667 |
| **MW0063595** | Sorbitan laurate | FA | Others | 7.17E-01 | 4884.02 | 196.1266667 |
| **MEDN0658** | Hexadecanedioic acid | Lipids | Free fatty acids | 7.32E-01 | 320246.9 | 6908.073333 |
| **MW0136588** | 6-{6-[2,3-dioxo-3-(2,4,6-trihydroxyphenyl)propyl]-2,3,4-trihydroxyphenoxy}-3,4,5-trihydroxyoxane-2-carboxylic acid | Organic acids | Organic acids | 6.47E-01 | 13479.16333 | 32060.08333 |
| **MW0155095** | Phe-Arg-Leu-Phe-Leu | Amino acids and derivatives | Amino acids and derivatives | 6.22E-01 | 84748.73 | 6323.42 |
| **MW0128962** | 4-Methoxy-9-[2-(sulfooxy)-3-methyl-3-hydroxybutoxy]-7H-furo[3,2-g][1]benzopyran-7-one | Organic acids | Organic acids | 5.90E-01 | 43103.88667 | 3319.873333 |
| **MW0158726** | Tyr-Pro-Phe | Amino acids and derivatives | Amino acids and derivatives | 5.68E-01 | 22581.87 | 3619.196667 |
| **MW0112732** | Isochlorogenic acid A | Phenolic acids | Phenolic acids | 9.44E-01 | 148244 | 61769.49333 |
| **MW0144202** | Abu-Asp-OH | Amino acids and derivatives | Amino acids and derivatives | 6.18E-01 | 28254.46333 | 1993.73 |
| **MW0145383** | Arg-Ile-Arg-Val-Met | Amino acids and derivatives | Amino acids and derivatives | 7.57E-01 | 2970.423333 | 725252.2233 |
| **MEDL02347** | L-Phenylalanine | Amino acids and derivatives | Amino acids and derivatives | 9.82E-01 | 118171.7333 | 23882.29 |
| **MEDP0160** | Adenosine | Nucleotides and derivatives | Nucleotides and derivatives | 9.79E-01 | 68642.44 | 17765.15 |
| **MEDL00391** | LysoPE 16:0 | Lipids | LPE | 9.46E-01 | 16059.47333 | 2463.4 |
| **MEDP0338** | LysoPC 16:0 | Lipids | LPC | 8.24E-01 | 30848.93333 | 4874.11 |
| **MW0166584** | N-[(5E)-2-oxo-4-sulfanyl-5-(sulfanylmethylidene)pyrrol-3-yl]acetamide | Alcohol and amines | Amines | 6.91E-01 | 333094.39 | 453.4433333 |
| **MW0158972** | Val-Gln-Met-Met-Thr | Amino acids and derivatives | Amino acids and derivatives | 8.34E-01 | 527.42 | 13630.31 |
| **MW0129016** | {2-[2-(3,4-dihydroxyphenyl)-4-[2-(3,4-dihydroxyphenyl)-3,5,7-trihydroxy-3,4-dihydro-2H-1-benzopyran-8-yl]-3,5,7-trihydroxy-3,4-dihydro-2H-1-benzopyran-6-yl]-3,5-dihydroxy-6-(hydroxymethyl)oxan-4-yl}oxidanesulfonic acid | Organic acids | Organic acids | 6.84E-01 | 159476.3567 | 2858657.64 |
| **MW0109333** | Pro-Pro-Arg | Amino acids and derivatives | Amino acids and derivatives | 9.03E-01 | 36911.00333 | 456.6166667 |
| **MW0159213** | Val-Tyr-Gln-Lys | Amino acids and derivatives | Amino acids and derivatives | 8.11E-01 | 6354.963333 | 323.87 |
| **MW0103634** | Nadide | Nucleotides and derivatives | Nucleotides and derivatives | 5.88E-01 | 222640.6833 | 112.1466667 |
| **MW0148733** | dTDP-4-(methylamino)-2,3,4,6-tetradeoxy-alpha-D-glucose | Nucleotides and derivatives | Nucleotides and derivatives | 5.54E-01 | 65193.57667 | 2331.86 |
| **MEDP0025** | L-Tryptophan | Amino acids and derivatives | Amino acids and derivatives | 9.55E-01 | 341335.5833 | 81710.89333 |
| **MW0155411** | Phe-Val-Lys-Lys | Amino acids and derivatives | Amino acids and derivatives | 9.34E-01 | 28304.36 | 108695.75 |
| **MEDL00392** | 1-(9Z-octadecenoyl)-sn-glycero-3-phosphocholine | Lipids | LPC | 8.57E-01 | 88932.47667 | 7946.41 |
| **MEDP0461** | 2-Methylguanosine | Nucleotides and derivatives | Nucleotides and derivatives | 9.24E-01 | 28921.54667 | 337.6466667 |
| **MEDP0495** | LPC(0:0/18:2) | GP | LPE | 9.51E-01 | 102184.12 | 14788.77333 |
| **MW0112620** | 3,4,5-trihydroxy-6-{[4-hydroxy-2,5-bis(hydroxymethyl)-2-{[3,4,5-trihydroxy-6-(hydroxymethyl)oxan-2-yl]oxy}oxolan-3-yl]oxy}oxane-2-carboxylic acid | Organic acids | Organic acids | 6.18E-01 | 63486.26 | 162207.29 |
| **MEDP1343** | LPC(0:0/18:3) | Lipids | LPC | 8.08E-01 | 466414.24 | 61812.31333 |
| **MW0145855** | Asn-Phe-Gly | Amino acids and derivatives | Amino acids and derivatives | 5.73E-01 | 33020.01333 | 2934.92 |
| **MW0054472** | (1S,2R,3R,4S,5R,6S,8R,9S,13S,16S,17R,18S)-11-ethyl-13-(hydroxymethyl)-4,6,16,18-tetramethoxy-11-azahexacyclo[7.7.2.12,5.01,10.03,8.013,17]nonadecane-8,9-diol | Alcohol and amines | Alcohols | 7.71E-01 | 10272.57667 | 47856.63 |
| **MW0144701** | Ala-Phe-Tyr | Amino acids and derivatives | Amino acids and derivatives | 6.77E-01 | 17441.69 | 4802.233333 |
| **MW0000266** | Galanthaminone | Alkaloids | Alkaloids | 6.65E-01 | 8404.12 | 2267.71 |
| **MW0118362** | 2-(9h-Carbazol-9-yl)benzoic acid | Organic acids | Organic acids | 7.13E-01 | 134710.1567 | 955327.8 |
| **MW0144387** | Adouetine Z | Amino acids and derivatives | Amino acids and derivatives | 6.17E-01 | 33919.80667 | 2701.336667 |
| **MW0153781** | Met-HoPhe-OH | Heterocyclic compounds | Heterocyclic compounds | 7.57E-01 | 67671.48667 | 496148.3633 |
| **MW0109106** | Phe-Glu-Arg | Amino acids and derivatives | Amino acids and derivatives | 6.96E-01 | 44647.24667 | 9011.273333 |
| **MW0103308** | 1-Deoxy-1-(7,8-dimethyl-2,4-dioxo-3,4-dihydro-2H-benzo[G]pteridin-1-ID-10(5H)-YL)-5-O-phosphonato-D-ribitol | Nucleotides and derivatives | Nucleotides and derivatives | 6.86E-01 | 2452.356667 | 33.85 |
| **MW0128532** | [3,4-Dihydroxy-5-[4-hydroxy-4-(hydroxymethyl)-3-sulooxyoxolan-2-yl]oxy-6-[5-hydroxy-2-(4-hydroxyphenyl)-4-oxochromen-7-yl]oxyoxan-2-yl]methyl acetate | Organic acids | Organic acids | 6.41E-01 | 29572.4 | 585.57 |
| **MW0158621** | Tyr-Thr-Gln-Arg | Amino acids and derivatives | Amino acids and derivatives | 6.59E-01 | 14547.77 | 186.55 |
| **MW0151503** | Ile-Lys-Ala-Arg | Amino acids and derivatives | Amino acids and derivatives | 8.28E-01 | 10841.77667 | 31274.96667 |
| **MW0134905** | 6-(3-{3,7-dihydroxy-4-[3,5,7-trihydroxy-2-(3-hydroxyphenyl)-3,4-dihydro-2H-1-benzopyran-8-yl]-3,4-dihydro-2H-1-benzopyran-2-yl}phenoxy)-3,4,5-trihydroxyoxane-2-carboxylic acid | Organic acids | Organic acids | 5.07E-01 | 79271.72333 | 4522.073333 |
| **MW0165939** | cyanidin 3-O-glucoside-7-O-(6-O-(p-hydroxybenzoyl)-glucoside) | Benzene and substituted derivatives | Benzene and substituted derivatives | 5.11E-01 | 3725.906667 | 146.2933333 |
| **MW0132726** | 3,5-Dihydroxy-2-(4-hydroxyphenyl)-7-[(3,4,5-trihydroxy-6-methyltetrahydro-2H-pyran-2-yl)oxy]-4H-1-benzopyran-4-one | Others | Ketone compounds | 7.61E-01 | 61761.78 | 289747.3333 |
| **MW0151998** | Kandelin A-1 | Flavonoids | Flavones | 6.15E-01 | 24098.75333 | 8996.603333 |
| **MW0128573** | [2-(methoxymethyl)-5-(3,5,7-trihydroxy-3,4-dihydro-2H-1-benzopyran-2-yl)phenyl]oxidanesulfonic acid | Organic acids | Organic acids | 6.29E-01 | 27897 | 665.37 |

**References**

1. X. Zheng, H. Lu, S. Feng, R. Hou, W. Liu, S. Ming, X. Xu, Y. Wu, and Z. Bo, “Effect of Polymer Molecular Weight and Processing Solvent on the Morphology and Photovoltaic Performance of Inverted Non‑Fullerene Solar Cells,” *Dyes and Pigments* 194 (2021): 109560.
2. M. Cloupeau and B. Prunet-Foch, “Electrohydrodynamic Spraying Functioning Modes: A Critical Review,” Journal of Aerosol Science 25 (1991): 1021–1036.

3. A. M. Gañán-Calvo, J. Dávila, and A. Barrero, “Current and Droplet Size in the Electrospraying of Liquids: Scaling Laws,” Journal of Aerosol Science 28 (1991): 249–275.

4. A. L. Yarin, S. Koombhongse, and D. H. Reneker, “Taylor Cone and Jetting from Liquid Droplets in Electrospinning of Nanofibers,” Journal of Applied Physics 90 (2001): 4836–4846.

5. S. Lin, X. Liu, J. Liu, H. Yuk, H.-C. Loh, G. A. Parada, C. Settens, J. Song, A. Masic, G. H. McKinley, and X. Zhao, “Anti-Fatigue-Fracture Hydrogels,” Science Advances 5 (2019): eaau8528.

6. T. Christoff-Tempesta, Y. Cho, D.-Y. Kim, M. Geri, G. Lamour, A. J. Lew, X. Zuo, W. R. Lindemann, and J. H. Ortony, “Self-Assembly of Aramid Amphiphiles into Ultra-Stable Nanoribbons and Aligned Nanoribbon Threads,” Nature Nanotechnology 16 (2021): 447–454.

7. E. Oliaei, P. Olsén, T. Lindström, and L. A. Berglund, “Highly Reinforced and Degradable Lignocellulose Biocomposites by Polymerization of New Polyester Oligomers,” Nature Communications 13 (2022): 5666.

8. X. Liang, G. Chen, I. M. Lei, P. Zhang, Z. Wang, X. Chen, M. Lu, J. Zhang, Z. Wang, T. Sun, Y. Lan, and J. Liu, “Impact-Resistant Hydrogels by Harnessing 2D Hierarchical Structures,” Advanced Materials 35 (2023): 2207587.

9. ASTM D882-18: Standard Test Method for Tensile Properties of Thin Plastic Sheeting, *ASTM International*, 2018.

10. Y. Yuan and T. R. Lee, “In Surface Science Techniques,” eds. G. Bracco and B. Holst, 3–34 (Springer, Berlin, Heidelberg, 2013).

11. F. S. Pantuso, M. P. Tolaba, and R. J. Aguerre, “A BET Approach to Multilayer Adsorption in Swelling Products,” Journal of Food Engineering 122 (2014): 68–73.

12. G. Kresse and D. Joubert, “From Ultrasoft Pseudopotentials to the Projector Augmented-Wave Method,” Physical Review B 59 (1991): 1758–1775.

13. J. P. Perdew, K. Burke, and M. Ernzerhof, “Generalized Gradient Approximation Made Simple,” Physical Review Letters 77 (1996): 3865–3868.

14. Y. Li, Z. Tang, W. Wang, X. Huang, Y. Lv, F. Qian, Y. Cheng, and H. Wang, “Improving Air Barrier, Water Vapor Permeability Properties of Cellulose Paper by Layer-by-Layer Assembly of Graphene Oxide,” Carbohydrate Polymers 253 (2021): 117227.

15. A. M. Adel, M. T. Al-Shemy, M. A. Diab, M. El-Sakhawy, R. G. Toro, L. Cerri, and D. Caschera, “Immobilization of TiO2 NP@ Oxidized Cellulose Nanocrystals for Paper-Based Active Packaging Materials,” International Journal of Biological Macromolecules 231 (2023): 123270.

16. S. A. A. Mohamed, M. El-Sakhawy, E. H. A. Nashy, and A. M. Othman, “Novel Natural Composite Films as Packaging Materials with Enhanced Properties,” International Journal of Biological Macromolecules 136 (2019): 774–781.

17. H. Chen, B. Wang, J. Li, G. Ying, and K. Chen, “High-Strength and Super-Hydrophobic Multilayered Paper Based on Nano-Silica Coating and Micro-Fibrillated Cellulose,” Carbohydrate Polymers 288 (2022): 119371.

18. R. Zhu, W. Lv, C. Sun, C. Qin, D. Zhang, and Z. Long, “A Facile Strategy to Fabricate High-Barrier, Water- and Oil-Repellent Paper with Carboxymethyl Cellulose/Collagen Fiber/Modified Polyvinyl Alcohol,” Carbohydrate Polymers 314 (2023): 120933.

19. G. Kale, T. Kijchavengkul, R. Auras, M. Rubino, S. E. Selke, and S. P. Singh, “Compostability of Bioplastic Packaging Materials: An Overview,” Macromolecular Bioscience 7 (2007): 255–277.

20. B. Balasubramaniam, P. Prateek, S. Ranjan, M. Saraf, P. Kar, S. P. Singh, V. K. Thakur, A. Singh, and R. K. Gupta, “Antibacterial and Antiviral Functional Materials,” ACS Pharmacology & Translational Science 4 (2021): 8–23.

21. M. Balouiri, M. Sadiki, and S. K. Ibnsouda, “Methods for in vitro evaluating antimicrobial activity: A review,” *Journal of Pharmaceutical Analysis* 6,2 (2016): 71–79.

22. U.S. Environmental Protection Agency (EPA), “Methods for Measuring the Acute Toxicity of Effluents and Receiving Waters to Freshwater and Marine Organisms,” 5th ed. Washington, DC: EPA Office of Water, 2002, EPA-821-R-02-012.

23. OECD, “Test No. 203: Fish, Acute Toxicity Test,” OECD Guidelines for the Testing of Chemicals, Section 2 (2019).

24. R. Z. Hu, L. Liu, E. J. Liu, J. Tu, X. H. Yao, P. Song, D. Y. Zhang, Z. H. Huang, and T. Chen, “Biomimetic Hierarchical Composites Inspired by Natural Pomelo Peel for Mechanical-Damage Resistance and Storage of Fruits,” Chemical Engineering Journal 485 (2024): 149853.

25. Y. Du, F. Yang, H. Yu, Y. Cheng, Y. Guo, W. Yao, and Y. Xie, “Fabrication of Novel Self-Healing Edible Coating for Fruits Preservation and Its Performance Maintenance Mechanism,” Food Chemistry 351 (2021): 129284.

26. Y. Chu, C. C. Gao, X. Liu, N. Zhang, T. Xu, X. Feng, X. Yang, X. Shen, and X. Tang, “Improvement of Storage Quality of Strawberries by Pullulan Coatings Incorporated with Cinnamon Essential Oil Nanoemulsion,” *Food Science and Technology* 122 (2020): 109054.

27. M. I. Pinzon, L. T. Sanchez, O. R. Garcia, R. Gutierrez, J. C. Luna, and C. C. Villa, “Increasing Shelf Life of Strawberries (Fragaria spp.) by Using a Banana Starch–Chitosan–Aloe Vera Gel Composite Edible Coating,” International Journal of Food Science & Technology 55 (2020): 92–99.

28. X. Zhou, G. Yin, Y. Huang, Y. Li, and D. Xie, “Biodegradable Nanofibrillated Cellulose/Poly(butylene Adipate-Co-Terephthalate) Composite Film with Enhanced Barrier Properties for Food Packaging,” Molecules 28 (2023): 2689.

29. S. F. Mirpoor, G. T. Patanè, I. Corrado, C. V. L. Giosafatto, G. Ginestra, A. Nostro, A. Foti, P. G. Gucciardi, G. Mandalari, D. Barreca, T. Gervasi, and C. Pezzella, “Functionalization of Polyhydroxyalkanoates (PHA)-Based Bioplastic with Phloretin for Active Food Packaging: Characterization of Its Mechanical, Antioxidant, and Antimicrobial Activities,” International Journal of Molecular Sciences 24 (2023): 11628.

30. T. Li, Y. Liu, Q. Qin, L. Zhao, Y. Wang, X. Wu, and X. Liao, “Development of Electrospun Films Enriched with Ethyl Lauroyl Arginate as Novel Antimicrobial Food Packaging Materials for Fresh Strawberry Preservation,” Food Control 130 (2021): 108371.

31. A. K. Chaudhari, S. Das, B. K. Singh, and N. K. Dubey, “Green Facile Synthesis of Cajuput (Melaleuca cajuputi Powell.) Essential Oil Loaded Chitosan Film and Evaluation of Its Effectiveness on Shelf-Life Extension of White Button Mushroom,” Food Chemistry 401 (2023): 134114.

32. M. Liu, H. Chen, F. Pan, X. Wu, Y. Zhang, X. Fang, X. Li, W. Tian, and W. Peng, “Propolis Ethanol Extract Functionalized Chitosan/Tenebrio molitor Larvae Protein Film for Sustainable Active Food Packaging,” Carbohydrate Polymers 343 (2024): 122445.

33. H. Chang, J. Xu, L. A. Macqueen, Z. Aytac, M. M. Peters, J. F. Zimmerman, T. Xu, P. Demokritou, and K. K. Parker, “High-Throughput Coating with Biodegradable Antimicrobial Pullulan Fibres Extends Shelf Life and Reduces Weight Loss in an Avocado Model,” Nature Food 3 (2022): 428–436.

34. Y. Yu, J. Zhou, Q. Chen, F. Xie, D. Zhang, Z. He, S. Cheng, and J. Cai, “Self-Reinforced Multifunctional Starch Nanocomposite Film for Litchi Fruit Postharvest Preservation,” Chemical Engineering Journal 486 (2024): 150262.

35. Ó. Ögmundarson, M. J. Herrgård, J. Förster, M. Z. Hauschild, and P. Fantke, “Addressing Environmental Sustainability of Biochemicals,” Nature Sustainability 3 (2020): 167–174.

36. Food and Agriculture Organization of the United Nations with Major Processing by Our World in Data, “Per Capita Consumption of Fruit,” FAO Data Sets (2024).

37. United Nations Population Fund, “World Population Dashboard Data Set” (2024).

38. European Environment Agency, “Forests, Health and Climate Change” (2011).

39. Lamberti, M., & Escher, F. "Aluminium Foil as a Food Packaging Material in Comparison with Other Materials," Food Reviews International, 23 (2007): 407–433.
